# Isothiocyanates Enhance the Anti-Melanoma Effect of Zebularine Through Modulation of Apoptosis and Regulation of DNMTs’ Expression, Chromatin Configuration and Histone Posttranslational Modifications Associated with Altered Gene Expression Patterns

**DOI:** 10.3390/epigenomes9010007

**Published:** 2025-02-25

**Authors:** Ioannis Anestopoulos, Ioannis Paraskevaidis, Sotiris Kyriakou, Louiza Potamiti, Dimitrios T. Trafalis, Sotiris Botaitis, Rodrigo Franco, Aglaia Pappa, Mihalis I. Panayiotidis

**Affiliations:** 1 Department of Cancer Genetics, Therapeutics & Ultrastructural Pathology, The Cyprus Institute of Neurology & Genetics, 2371 Nicosia, Cyprus; ioannisa@cing.ac.cy (I.A.); sotirisk@cing.ac.cy (S.K.); louizap@cing.ac.cy (L.P.); 2 Perelman School of Medicine, University of Pennsylvania, Philadelphia, PA 19104, USA; ioannis.paraskevaidis@penn.medicine.upenn.edu; 3 Laboratory of Pharmacology, Medical School, National & Kapodistrian University of Athens, 11527 Athens, Greece; dtrafal@med.uoa.gr; 4 Department of Surgery, School of Medicine, University Hospital, Democritus University of Thrace, 68100 Alexandroupolis, Greece; smpotait@med.duth.gr; 5 School of Veterinary Medicine & Biomedical Sciences, University of Nebraska-Lincoln, Lincoln, NE 68583, USA; rodrigo.franco@unl.edu; 6 Redox Biology Centre, University of Nebraska-Lincoln, Lincoln, NE 68583, USA; 7 Department of Molecular Biology & Genetics, Democritus University of Thrace, 68100 Alexandroupolis, Greece; apappa@mbg.duth.gr; 8 Department of Comparative Biomedical Sciences, School of Veterinary Medicine, Mississippi State University, Starkville, MS 39762, USA

**Keywords:** melanoma, zebularine, isothiocyanates, apoptosis, DNMTs, lysine acetylation, lysine methylation

## Abstract

**Background:** In the present study, we aimed to characterize the cytotoxic efficacy of Zebularine either as a single agent or in combination with various isothiocyanates in an in vitro model consisting of human melanoma (A375, Colo-679) as well as non-tumorigenic immortalized keratinocyte (HaCaT) cells. **Methods:** In this model, we have evaluated the anti-melanoma effect of Zebularine (in single and combinatorial protocols) in terms of cell viability, apoptotic induction and alterations in ultrastructural chromatin configuration, protein expression levels of DNA methyltransferases (DNMTs) and associated histone epigenetic marks capable of mediating gene expression. **Results:** Exposure to Zebularine resulted in dose- and time-dependent cytotoxicity through apoptotic induction in malignant melanoma cells, while neighboring non-tumorigenic keratinocytes remained unaffected. A more profound response was observed in combinational protocols, as evidenced by a further decline in cell viability leading to an even more robust apoptotic induction followed by a differential response (i.e., activation/de-activation) of various apoptotic genes. Furthermore, combined exposure protocols caused a significant decrease of DNMT1, DNMT3A and DNMT3B protein expression levels together with alterations in ultrastructural chromatin configuration and protein expression levels of specific histone modification marks capable of modulating gene expression. **Conclusions:** Overall, we have developed a novel experimental approach capable of potentiating the cytotoxic efficacy of Zebularine against human malignant melanoma cells while at the same time maintaining a non-cytotoxic profile against neighboring non-tumorigenic keratinocyte (HaCaT) cells.

## 1. Introduction

Melanoma tumorigenesis and progression have been linked to epigenetic modifications such as aberrant DNA methylation patterns, histone modifications and alterations of non-coding RNA profiles. Specifically, an altered epigenetic landscape has been associated with abnormal gene expression patterns further contributing to abnormal proliferation rates, tolerance to current therapies, apoptotic evasion, cell plasticity, increased invasiveness and migration, thus leading to melanoma occurrence and progression [1,2,3,4,5]. In this context, targeting (and potentially reversing) deregulated epigenetic mechanisms has emerged as an attractive therapeutic strategy in malignant melanoma management [6,7]. One of the best-characterized epigenetic modifications is DNA methylation, a modification catalyzed by the activity of DNA methyltransferases (DNMTs), which are categorized as either de novo (e.g., DNMT3A and DMT3B) or maintenance (e.g., DNMT1), catalyzing either the methylation of un-methylated DNA or semi-methylated DNA, respectively. Interestingly, DNA methylation occurs in genomic areas with a high density of cytosine-guanine dinucleotides (CpG islands) observed either in promoter sequences or dispersed across the human genome [8,9,10]. Generally, abnormal patterns of DNA methylation are related to gene silencing and include either hypermethylation of gene promoters or reduced global (hypo) methylation, resulting in abnormal expression of genes essential for the multistage carcinogenic process. In particular, site-specific hypermethylation is associated with the silencing of tumor suppressor genes (TSGs) involved in important cellular processes leading to tumor occurrence [11,12]. To this end, melanoma onset and progression have been associated with promoter hyper-methylation and silencing of tumor suppressor genes, including *PTEN*, *p16/14*, *RASSF1A*, *MGMT*, *E-cadherin*, *CDKN1B*/*2A* and *ARF*, among others, all of which are involved in cellular processes such as proliferation, cell cycle regulation, apoptosis, DNA repair and metastasis. Similarly, the hypomethylation of *LINE-1*, *MAGE-1/2/3/4* and *TKTL1* have been associated with melanoma progression and increased metastatic potential [1,2,9,13]. In this context, different types of DNMT inhibitors (DNMTi) have been developed, including the nucleoside analog of cytidine, called Zebularine (ZEB), an epigenetic drug compound which is more stable and less toxic compared to the previously developed demethylating agents including 5-azacytidine [5-azaC; Azacytidine] and its deoxy derivative 5-aza-2′-deoxycytidine [5-aza-dC; Decitabine]. All these drug compounds are known to exert their function through interaction and formation of a stable complex with DNMTs, thus inhibiting their enzymatic activity and ultimately leading to reduced methylation levels [14,15,16].

On the other hand, isothiocyanates (ITCs) are sulfur-containing phytochemicals found in cruciferous vegetables and derived from the hydrolysis of glucosinolates by myrosinase. Major, and most well-described, types of ITCs include Iberin (IBN), Benzyl-ITC (BITC), Allyl-ITC (AITC), Sulforaphane (SFN) and Phenethyl-ITC (PEITC). These have been shown to exert considerable anti-neoplastic activity against different types of cancers through cell cycle arrest, apoptosis, autophagy, oxidative stress, inhibition of invasiveness and angiogenesis in both in vitro and in vivo models [17,18,19]. Additionally, different ITCs have been reported to exert their anti-melanoma properties via regulation of the epigenetic machinery by altering the levels of specific histone acetylation and methylation marks in addition to modifying the expression and activity levels of key histone deacetylases (HDACs), acetyltransferases (HATs), methyltransferases (HMTs) and DNMTs [20,21,22,23,24].

Although different types of epigenetic drugs have been reported to exhibit significant anticancer activity against various hematological malignancies, such effect is rather limited against solid tumors, mainly due to a lack of specificity associated with high rates of toxicity and development of tumor resistance [25,26]. In this context, natural compounds and dietary phytochemicals have been either used or considered for use in cancer treatment. For instance, various studies have documented their ability to attenuate cancer progression as epigenetic modulators by targeting critical epigenetic pathways. In addition, their strong antioxidant activity (accompanied by low levels of toxicity) suggests that they could emerge as potentially promising adjuvant compounds in combinatorial treatments with epigenetic drugs toward a more efficient cancer therapeutic management [20,27].

In this study, we aimed to characterize the anti-melanoma effect of ZEB using an in vitro model of human malignant melanoma consisting of melanoma (A375 and Colo-679) as well as neighbouring non-tumorigenic immortalized keratinocyte (HaCaT) cells. Furthermore, we aimed to investigate the ability of SFN, IBN and BITC to potentiate ZEB’s anti-melanoma efficacy by inducing the apoptotic and epigenetic response pathways as underlined mechanism(s) of its action.

## 2. Results

### 2.1. Determination of ZEB-Induced Cytotoxicity in Human Malignant Melanoma Cells

**Figure 1:**
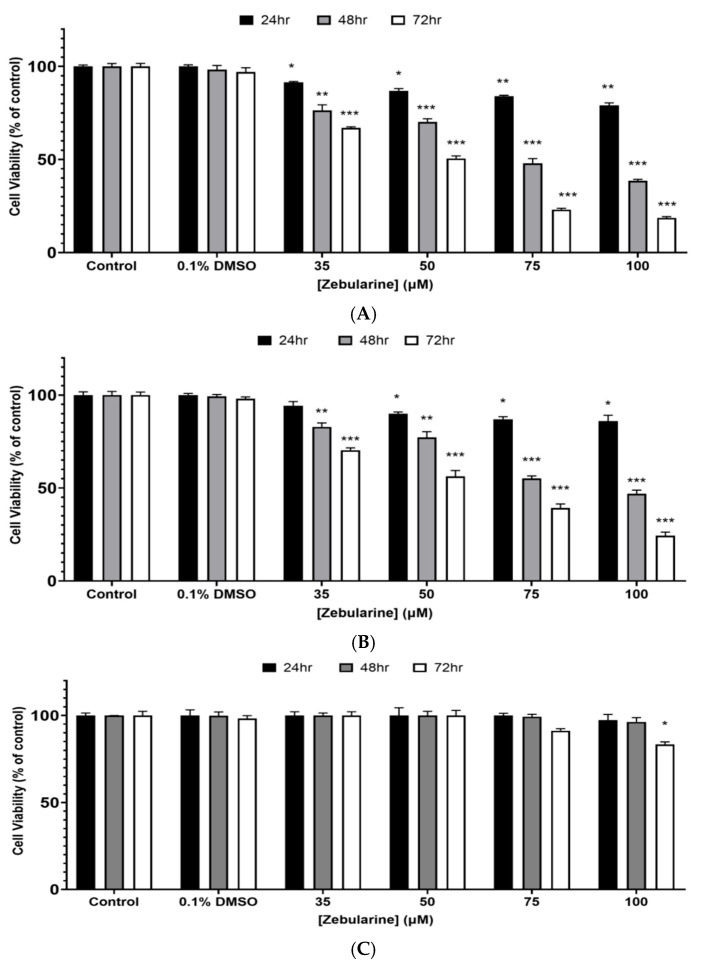
Cytotoxic profile of ZEB against melanoma and keratinocyte cells. Human malignant melanoma A375 (**A**), Colo-679 (**B**) and non-malignant immortalized keratinocyte HaCaT (**C**) cells were exposed to increasing concentrations of ZEB (35–100 μΜ) for 24–72 h. Cell viability was determined using the Alamar Blue assay. All data are expressed as means ± SEM and are representative of three independent experiments. Statistical significance is indicated by * *p* < 0.05, ** *p* < 0.01 and *** *p* < 0.001 relative to corresponding 0.1% DMSO controls.

**Figure 2:**
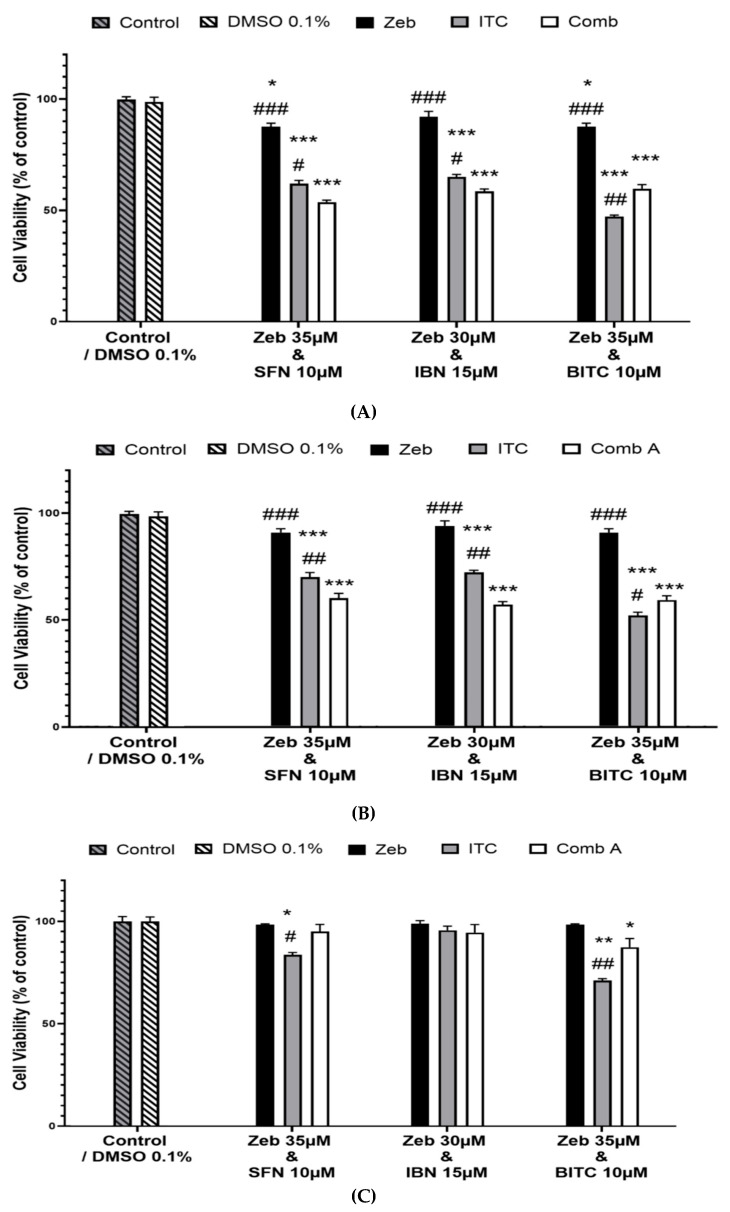
Cytotoxic profile of selected single and combinatorial exposures to ZEB with each ITC. Human malignant melanoma A375 (**A**), Colo-679 (**B**) and non-malignant immortalized keratinocyte HaCaT (**C**) cells were exposed to either ZEB or each ITC alone or to combined exposures between ZEB and each ITC. Cell viability was determined using the Alamar Blue assay. All data are expressed as means ± SEM and are representative of three independent experiments. Statistical significance is indicated by * *p* < 0.05, ** *p* < 0.01 and *** *p* < 0.001 relative to corresponding 0.1% DMSO controls and # *p* < 0.05, ## *p* < 0.01 and ### *p* < 0.001 relative to corresponding combinatorial exposures.

Cells were exposed to a range of ZEB concentrations (0–100 μM) over 24–72 h. The data analysis revealed that ZEB induced significant dose- and time-dependent cytotoxicity against A375 cells. Specifically, this effect was observed as early as 48 h and was even further potentiated at 72 h of exposure. This pattern of cytotoxicity was particularly evident at the highest concentrations of ZEB (Figure 1A). In parallel, Colo-679 cells appeared to be slightly more resistant but, nevertheless, showed the same pattern of cytotoxicity as that observed in A375 cells (Figure 1B). Finally, ZEB exerted no cytotoxicity against HaCaT cells, with an exception only at 72 h of exposure and at the highest concentration of ZEB (Figure 1C). All EC_50_ values of ZEB were calculated in both melanoma cell lines. Overall, it was evident that, as the time of exposure was increased, ZEB was becoming more toxic, a pattern followed in both cell lines (Table 1). Next, we sought to determine the combinatorial cytotoxic effect of ZEB with each of the ITCs. Specifically, we determined the cytotoxic potential of an optimized protocol of combinatorial exposures: (i) 35 μΜ ZEB/10 μM SFN, (ii) 30 μΜ ZEB/15 μM IBN and (iii) 35 μΜ ZEB/10 μM BITC The rationale for the selection of all the above-indicated optimized co-exposure conditions was based on their efficacy to sustain cytotoxicity in A375 cells (at their EC_50_ values) while at the same time maintaining viability of HaCaT cells at untreated (control) levels (Table S1; Figures S1 and S2). It was evident that combined exposures to ZEB with each of the ITCs resulted in potentiation of cytotoxicity, with differences being more substantial when compared to ZEB alone than to each of the ITCs individually. This was the case for both A375 (Figure 2A) and Colo-679 cells (Figure 2B), while HaCaT cells appeared to remain largely unaffected (Figure 2C).

### 2.2. Combination Index Analyses for Determination of Co-Exposure Interactions Type

After the determination of cytotoxicity, we determined the nature of the interaction between ZEB and each ITC in A375 cells by a combination index analysis (CI). Following isobolographic analyses, it was observed that the interactions between ZEB with each ITC were either synergistic (CI < 1) or antagonistic (CI > 1) according to the relevant experimental condition. Specifically, co-exposures of ZEB with IBN or BITC were found to be antagonistic, while that of ZEB with SFN was synergistic (Table S2 and Figure S3).

### 2.3. Determination of Caspase-3 Activity as an Index of Apoptotic Induction

**Figure 3:**
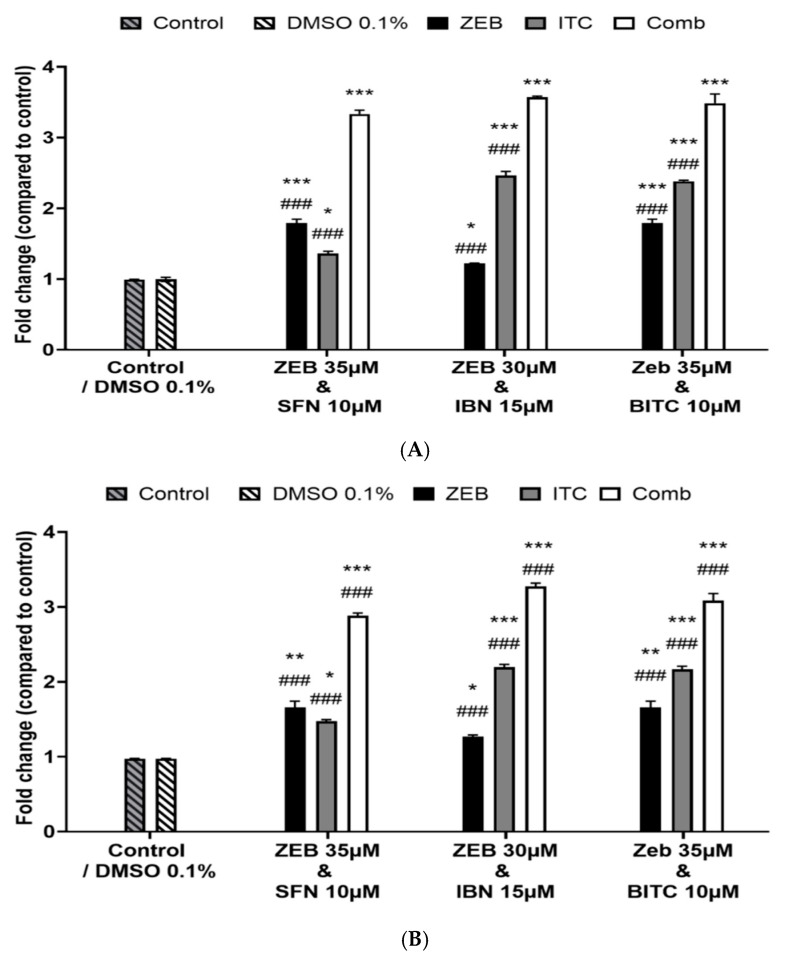
Determination of caspase-3 activity levels in human malignant melanoma cells. A375 (**A**) and Colo-679 (**B**) cells were exposed to either ZEB or each ITC alone or to combined exposures between ZEB and each ITC for 72 h. Caspase-3 enzymatic activity was determined using a commercial fluorometric multiplex assay kit. All data are expressed as means ± SEM and are representative of three independent experiments. Statistical significance is indicated by * *p* < 0.05, ** *p* < 0.01 and *** *p* < 0.001 relative to corresponding 0.1% DMSO controls and # *p* < 0.05, ## *p* < 0.01 and ### *p* < 0.001 relative to corresponding combinatorial exposures.

Next, we investigated the effect of the optimized combinatorial exposure protocol on the activity levels of caspase-3 against A375 and Colo-679 cells (Figure 3A,B). Our data revealed that Caspase-3 activity was significantly increased in both A375 (Figure 3A) and Colo-679 (Figure 3B) cells when each compound was compared to the control or when the combinatorial exposure was compared with each of the compounds alone.

### 2.4. Profiling the Response of Apoptosis-Related Genes After Different Exposure Conditions

Then, we evaluated by RT-PCR the effect of ZEB in regulating the expression of several genes involved in intrinsic and extrinsic apoptotic pathways, as well as in various anti-apoptotic pathways. The selection criteria included up- or down-regulation values higher than 0.5-fold in at least two out of three co-exposure conditions in both A375 and Colo-679 cells. Overall, a number of intrinsic (*CASP3*, *BAX, CYCS*), extrinsic (*FADD, CASP8*, *TNFRSF10B*, *TNFRSF10A*) and anti-apoptotic (*TNFRSF10D*, *TNFRSF10C*) genes were shown to be differentially regulated following co-exposure conditions with ZEB and each of the ITCs in both A375 and Colo-679 cells (Tables S4 and S5).

### 2.5. The Effect of Different Exposure Conditions on DNMTs’ Protein Expression Levels

**Figure 4:**
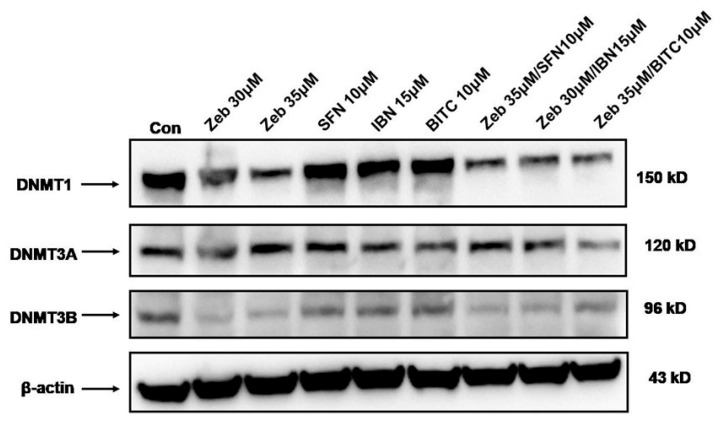
The effect of ZEB and each ITC alone or in combined exposures to ZEB with each ITC on protein expression levels of DNMTs. A375 cells were treated with either ZEB (30 μΜ/35 μΜ) or each ITC (10 μM SFN, 15 μM IBN and 10 μΜ BITC) alone for 72 h or, in combinatorial exposures, pre-treated with ZEB (30 μΜ/35 μΜ), for 24 h, followed by addition of SFN (35 μΜ ZEB/10 μΜ SFN), IBN (30 μΜ ZEB/15 μΜ IBN) and BITC (35 μΜ ZEB/10 μΜ BITC) for an additional 48 h (72 h total). Whole cell extracts were analyzed for DNMT1, DNMT3A and DNMT3B protein expression levels. The western blot shown is representative of two independent experiments. Equal protein loading was verified by stripping and re-probing the same membranes with β-actin.

In this set of experiments, we evaluated the effect of ZEB alone or in combination with each ITC on protein expression levels of DNMT1, DNMT3A and DNMT3B in A375 cells. Western immunoblotting analyses revealed significant changes in the expression levels of DNMT1 and DNMT3B when ZEB alone was compared to the control. However, this was not the case when each of the ITCs alone was compared to the control, as there was no significant change in the protein expression levels of DNMTs (Figure 4). However, in the case of combinatorial exposure conditions, all three DNMTs exhibited reduced protein expression levels. This was particularly evident in co-exposure of ZEB with BITC, which further reduced protein expression levels of DNMT1 and DNMT3A compared to ZEB alone (Figure 4).

### 2.6. The Effect of Different Exposure Conditions on Chromatin Organization

**Figure 5:**
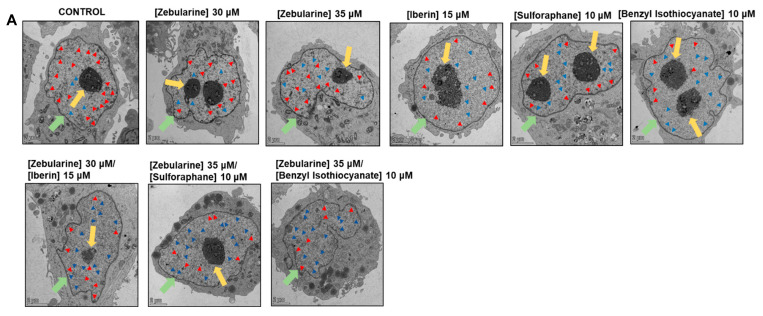

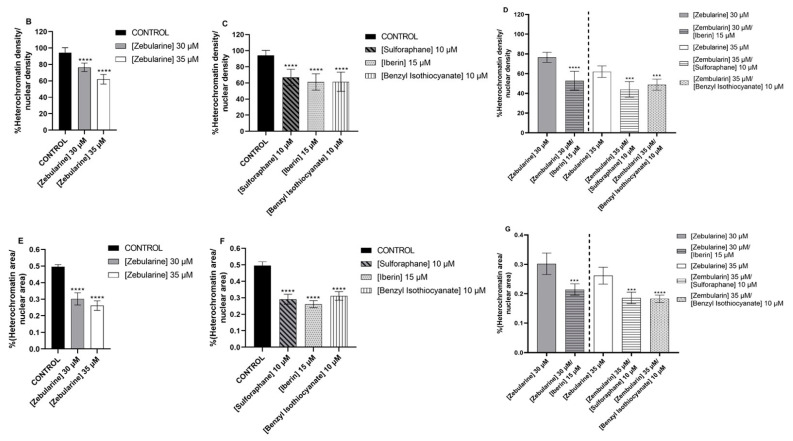
The effect of ZEB and each ITC alone or in combined exposures to ZEB with each ITC on chromatin state/organization. Representative electron micrographs were obtained from ultra-thin sections (0.1 μm) after exposures of A375 cells to either ZEB (30 μΜ/35 μΜ) or each ITC (10 μM SFN, 15 μM IBN and 10 μΜ BITC) alone for 72 h. In combinatorial exposures, A375 cells were pre-treated with ZEB (30 μΜ/35 μΜ) for 24 h, followed by addition of SFN (35 μΜ ZEB/10 μΜ SFN), IBN (30 μΜ ZEB/15 μΜ IBN) and BITC (35 μΜ ZEB/10 μΜ BITC) for an additional 48 h (72 h total). Red arrows indicate heterochromatin compact sites (**A**), whereas blue arrows point euchromatin clusters. The nucleus and nucleoli are indicated with green and yellow arrows, respectively. Heterochromatin density/nuclear density (% of control) of A375 cells exposed to ZEB or each ITC alone or in combinatorial exposures to ZEN with each ITC (**B**–**D**). Heterochromatin area/nuclear area (% of control) of A375 cells exposed to ZEB or each ITC alone or in combinatorial exposures between ZEN with each ITC (**E**–**G**). Overall, 20 micrographs were analyzed through ImageJ software, and data are expressed as means ± SEM. In brief, nuclear and heterochromatin area and density were measured by analyzing parameters such as integrated density, min and max grey value, mean grey value and total area. Heterochromatin areas: tightly packed chromatin areas with low accessibility of transcription factors and, thus, transcriptionally inactive. Euchromatin areas: loosely packed chromatin areas, accessible to transcriptional factors and, thus, associated with transcriptional activity. Statistical significance is indicated by *** *p* < 0.001 and **** *p* < 0.0001 relative to corresponding controls.

By utilizing transmission electron microscopy (TEM), we aimed to determine specific ultrastructural modifications, including heterochromatin density/nuclear density as well as heterochromatin area/nuclear area. To this end, A375 cells were treated with either ZEB alone or with each of the ITCs for 72 h. Electron micrographs were obtained, and quantification of these ultrastructural alterations was performed by ImageJ software, 1.54g. Data analyses revealed that exposures of A375 cells to ZEB caused a significant reduction of the heterochromatin density/nuclear density ratio, indicating an important heterochromatin de-condensation (less dense chromatin areas/more relaxed chromatin configuration, accessible to transcription factors) (Figure 5A,B), while a similar effect was also observed after exposure to each ITC (Figure 5A,C). On the other hand, co-exposures to ZEB with each ITC further potentiated this effect when compared to ZEB alone, thereby suggesting an even more significant heterochromatin de-condensation (Figure 5A,D). Moreover, this pattern of de-condensation was similar when tested for heterochromatin area/nuclear area ratio (Figure 5A,E–G). Accordingly, we observed an increase in the total numbers of euchromatin areas/clusters, especially after co-exposures to ZEB with each of the ITCs (Figure 5, blue arrows). Specifically, this pattern was accompanied by less dense heterochromatin areas (more relaxed chromatin configuration), mainly after exposures with each ITC alone, as well as in co-exposures with ZEB as opposed to either control (untreated cells) or A375 cells exposed to both concentrations of ZEB (Figure 5, red arrows). Overall, co-exposures to ZEB with ITCs indicate a transition from heterochromatin to euchromatin configuration (thus, more accessible to transcription factors), thereby favoring increased transcriptional activity and ultimately recapitulating, at least in part, our data relevant to their effect on inducing apoptotic gene expression levels.

### 2.7. The Effect of Different Exposure Conditions on Protein Expression Levels of Histone Modifications Associated with Chromatin Organization

**Figure 6:**
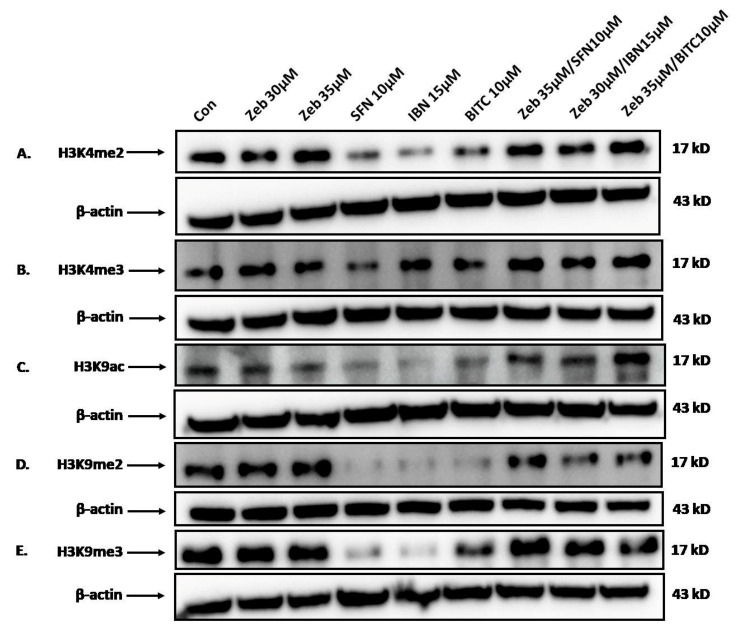
The effect of ZEB and each ITC alone or in combined exposures to ZEB with each ITC on protein expression levels of H3K4me2/3 (di-/tri-methylation of histone H3 at Lys4) (**A**,**B**), H3K9ac (acetylation of histone H3 at Lys9) (**C**) and H3K9me2/3 (di-/tri-methylation of histone H3 at Lys9) (**D**,**E**). A375 cells were treated with either ZEB (30 μΜ/35 μΜ) or each ITC (10 μM SFN, 15 μM IBN and 10 μΜ BITC) alone for 72 h or, in combinatorial exposures, pre-treated with ZEB (30 μΜ/35 μΜ) for 24 h, followed by addition of SFN (35 μΜ ZEB/10 μΜ SFN), IBN (30 μΜ ZEB/15 μΜ IBN) and BITC (35 μΜ ZEB/10 μΜ BITC) for an additional 48 h (72 h total). Whole cell extracts were analyzed for H3K9ac, H3K4me2/3, H3K9ac and H3K9me2/3 protein expression levels. The western blots shown are representative of two independent experiments. Equal protein loading was verified by stripping and re-probing the same membranes with β-actin.

Finally, we assessed the effect of ZEB alone or in co-exposures with each ITC on the expression levels of various histone modifications associated with either euchromatin and gene activation (i.e., di- and/or tri-methylation of histone H3 at Lys4 [H3K4me2/3] and/or acetylation of histone H3 at Lys9 [H3K9ac]) or heterochromatin and, thus, gene repression (di- and/or tri-methylation of histone H3 at Lys9 [H3K9me2/3]). Our data indicated an increase of H3K4me2 levels after exposure to the highest concentration of ZEB alone, while a significant reduction was observed after exposure to each ITC. Co-exposures to ZEB with each ITC increased the levels of H3K4me2 to those comparable with ZEB alone (Figure 6A). Similarly, the same pattern was observed in the levels of H3K4me3, except that the reduction of these levels was not as significantly reduced after exposure to each ITC (Figure 6B). In addition, the levels of H3K9ac were significantly reduced after exposure to each ITC alone, while co-exposure conditions induced a significant induction in its levels, especially in the case of BITC (Figure 6C). The same pattern was observed in H3K9me2 levels, except that when exposed to each ITC, these levels were almost reduced to zero. Under co-exposure conditions, these levels were significantly increased again but never reached those observed with either concentration of ZEB alone (Figure 6D). Finally, H3K9me3 levels appeared almost unaffected after exposure to each concentration of ZEB alone, while exposure to each ITC almost completely inhibited these levels (with the exception of BITC). Once again, co-exposure conditions significantly increased these levels back to those observed during exposure to either concentration of ZEB alone (Figure 6E). Overall, our findings suggest a pretty consistent observation where exposure to each ITC alone significantly inhibits the expression levels of each of the examined posttranslational modifications, whereas co-exposure conditions were capable of reverting these levels (to a variable extent) back to those observed under exposure to each concentration of ZEB alone.

## 3. Discussion

Various reports have indicated that the deregulation of epigenetic mechanisms, beyond genetic alterations, is a critical factor further contributing to the onset and development of various pathological conditions, including cancer, in terms of structural dynamics of chromatin (accessibility-compactness) and regulation of the expression of different genes implicated in carcinogenesis [28,29]. Given the reversible nature of the epigenetic alterations, epigenetic therapy by means of epi-drugs is a promising anticancer therapeutic approach [30,31,32,33,34,35]. However, only a limited number of epi-drugs have been clinically utilized (mainly against hematological malignancies) since their efficacy against solid tumors was shown to be limited, mainly due to their reduced activity and/or unfavorable toxicity [33,36]. On the other hand, various studies have indicated the important role of phytochemicals as promising preventive and/or suppressive agents of the carcinogenic process through epigenetic regulation of tumor suppressor genes and oncogenes [37]. Specifically, ITCs have attracted scientific interest based on their unique properties, toward cancer prevention and treatment, mediated through a variety of mechanisms including (i) induction of apoptotic cell death and cell cycle arrest, (ii) inhibition of metastasis and angiogenesis and, interestingly, (iii) exhibition of epigenetic modulatory properties targeting all types of epigenetic modifications [38]. In this context, combined treatment protocols between various phytochemicals (capable of regulating the epigenetic machinery) with various epi-drugs appear as a promising and safer therapeutic approach in minimizing potential side effects while maintaining or even enhancing their anti-cancer efficacy [39,40].

Our results revealed that ZEB exhibited considerable cytotoxic activity against A375 and Colo-679 (although to a lesser extent) malignant melanoma cells while exerting no cytotoxicity against neighboring non-tumorigenic keratinocytes. Several reports have indicated the anti-proliferative activity of this epi-drug against various types of cancer, such as in myeloma [41] and hepatoma [42]. However, reports on the cytotoxic activity of ZEB in melanoma are rather limited. To this end, we have shown that co-exposures of melanoma cells to different types of ITCs (SFN, IBN and BITC) with ZEB potentiate the anti-melanoma effect of the epi-drug to a variable extent. In addition, when assessing the nature of the interaction between ITCs and ZEB, the Combination Index analysis (CI) revealed the following three distinct patterns: (i) a synergistic effect between ZEB and SFN, (ii) an additive effect between ZEB and IBN and (iii) an antagonistic effect between ZEB and BITC. The underlying mechanism(s) by which co-exposures of ZEB with different ITCs results in distinct patterns of CI analysis warrants further investigation. However, synergisms between different ITC chemical structures with higher rates of cytotoxicity are characterized by synergistic interactions indicative of the observed CI patterns (especially in co-exposures between ZEB and SFN). To this end, we have previously shown a similar synergistic effect with co-exposures to Tazemetostat (an EZH2 inhibitor) and SFN in A375 melanoma cells, thus further supporting SFN’s anticancer potential in combinatorial treatment protocols with various epi-drugs (e.g., Zebularine and Tazemetostat) [43]. Furthermore, we revealed that all ITCs were capable of potentiating the apoptotic effect of ZEB by inducing caspase-3 activation. Moreover, this finding was accompanied by a further increase of expression levels of various apoptotic genes implicated in intrinsic and extrinsic apoptotic as well as anti-apoptotic cascades. Apoptotic induction as an anti-cancer mechanism of the action of ZEB and ITCs has been previously reported through a number of pleiotropic underlined mechanisms involved in various cancer models, like hepatoma [44], breast carcinoma [45], cholangiocarcinoma [42], glioblastoma [46,47], ovarian [48] and lung [49] carcinomas, as well as gastric adenocarcinoma [50].

Abnormal DNA methylation has emerged as a key hallmark in melanoma pathogenesis and progression, thus appearing as a promising target for anti-cancer therapy. For example, the depletion of DNMT1 in human melanoma cells led to hypomethylation and re-expression of the germ line-specific *MAGE-A1* transgene, commonly suppressed in melanoma. In addition, the depletion of DNMT3A in mouse melanoma cells inhibited tumor growth and metastasis in a xenograft model, while DNMT3B increased proportionally with melanoma progression, leading to the silencing of tumor suppressor gene *p16INK4A* [51,52]. In addition, different reports indicate an association between an abnormal epigenetic landscape and resistance to both targeted and immunotherapeutic agents in malignant melanoma. Specifically, a major role of altered expression levels of DNMTs has been described in the development of resistance to temozolomide (TMZ) through the silencing of the DNA mismatch repair gene *MLH1*, while increased levels of DNMT1 have been associated with Vemurafenib-resistant tumors among stage IV melanoma patients [53,54,55]. Our data indicated that exposure to ZEB caused a reduction primarily in DNMT1 and DNMT3B protein expression levels, an effect which was reversed upon the action of all ITCs alone, which increased back to control levels the expression of these DNMTs. However, combined exposures to ZEB with each ITC resulted in a further decrease of DNMT1 and DNMT3A expression levels compared to the effect of ZEB alone. Different classes of ITCs have been reported to exert their anti-cancer activity through the modulation of distinct epigenetic processes (including alterations in DNMT expression patterns) in various cancer models like prostate [56,57,58] and breast [59] carcinomas. In this context, it would be of benefit to explore if the reduction in protein expression levels of different classes of DNMTs (following co-treatments between ZEB and various ITCs) could also reverse the resistance of melanoma cells to targeted and/or immunotherapeutic agents.

DNA methylation can induce the formation of a heterochromatin state characterized by a closed/dense chromatin configuration known to be transcriptionally inactive, thus leading to gene silencing [60]. Our data revealed that co-exposures to each ITC were capable of potentiating the effect of ZEB alone, causing a decrease of chromatin compaction in terms of chromatin de-condensation, as we have observed a potent reduction in ultrastructural modifications such as (i) heterochromatin density/nuclear density and (ii) heterochromatin area/nuclear area. Overall, the above findings indicate a more relaxed chromatin configuration, as evidenced by a higher number of euchromatin areas in A375 cells after combined exposures to ZEB with various ITCs, thereby suggesting an increase in transcriptional activity. In addition, such response patterns may recapitulate, at least in part, the differential effect of co-exposures on apoptotic gene expression and potential re-activation of previously silenced tumor suppressor genes. In this context, there are very limited studies suggesting a direct effect of ZEB exposure on heterochromatin de-condensation and heterochromatin amount. However, some studies have reported on the capacity of ZEB to efficiently decrease DNA methylation through partial de-condensation of heterochromatin and reduced heterochromatin amount in *Arabidopsis thaliana* plant [61,62].

On the other hand, there is strong evidence that DNA methylation is associated with various types of histone post-translational modifications (PTMs) (e.g., methylations and acetylations) capable of inducing alterations in gene expression. Specifically, there seems to be a bidirectional connection where DNA methylation can directly affect the methylation states of neighboring histone residues, while histone lysine methylation marks can directly influence DNA methylation patterns. In this context, different types of histone PTMs were identified as characteristic features of either euchromatin or heterochromatin configuration. While histone acetylation is associated with transcriptional activity, methylation of histone residues can cause either gene activation or repression, depending on the specific residue that is modified [63,64]. For example, acetylation marks of histone H3 at either Lys9 (H3K9ac) and/or Lys27 residues (H3K27ac) are associated with a more relaxed (euchromatin) configuration and, thus, gene transcription [65,66], while marks of di- and/or tri-methylation of either histone H3 at Lys4 (H3K4me2/3) and/or Lys9 residues (H3K9me2/3) are related to euchromatin and heterochromatin configuration, respectively, thus differentially regulating gene activity [67,68]. For instance, while H3K4me2 and H3K4me3 have been reported as markers of active genes, they have distinct roles since elevated levels of H3K4me2 are found at the 5′ end of transcribing genes while promoters of actively transcribed genes are characterized by the presence of H3K4me3 marks [69,70]. In the same context, it has been proposed that H3K4me3 marks can block de novo methylation [71], and although the exact mechanism is not fully understood, it has been reported that either the presence of H3K9me3 marks can recruit DNMT3A/DNMT3B or DNMTs can associate/interfere with H3K9 methyltransferases (i.e., SUV39H1/2) to methylate heterochromatin regions rich in H3K9 marks, thus promoting de novo DNA methylation and further contributing to condensed chromatin configuration and gene repression [72,73]. Our western blot results indicated that in the majority of combined exposures to ZEB with each ITC, protein expression levels of H3K4me2/3 were further induced, thus indicating a more relaxed chromatin configuration and the potential induction of transcriptional activity. This finding was further substantiated by the increased levels of H3K9ac, another important signature mark descriptive of actively transcribed genes. In this context, the reduction of H3K9me2 marks suggests a more potent de-condensation of heterochromatin, a finding that is in accordance with TEM analysis. Work from our group has characterized ITCs as important epigenetic modulators since their anticancer activity was reported to be mediated, at least in part, through the regulation of abnormal histone dynamic patterns [43].

## 4. Conclusions

Overall, we have developed an optimized experimental therapeutic protocol based on novel combinatorial treatments between ZEB and different ITCs, providing evidence of its anti-cancer efficacy. To the best of our knowledge, combinatorial studies between epi-drugs and different classes of ITCs against melanoma are rather limited, thus further highlighting the novelty of our study. Specifically, through our experimental platform, we were capable of enhancing the anti-melanoma activity of ZEB through co-exposures to ITCs through apoptotic induction while at the same time maintaining a non-cytotoxic profile against neighboring non-tumorigeic keratinocytes. Furthermore, such an anti-melanoma effect appeared to be mediated by significant alterations in the protein expression levels of major DNMTs, along with histone modification marks and alterations in chromatin configuration associated with the modulation of gene expression.

However, more elaborate experiments are needed to better characterize the underlying molecular mechanism(s) of such an anti-melanoma approach. To this end, the application of artificial intelligence (AI) analysis algorithms [i.e., network-based and/or machine learning-based (ML) biology analysis algorithms] that integrate and process multi-omics data could further aim to identify new targets of our experimental protocol [74,75]. Moreover, the use of either patient-derived xenograft (PDX) and/or patient-derived organoid (PDO) cultures (which maintain the major properties of native tumors) obtained from biobank repositories appears to be an effective approach for validating the therapeutic efficacy of our experimental platform. In addition, AI-based prediction of patients’ response against novel therapeutic schemes could ultimately provide a basis for future clinical trials in melanoma therapy [75,76,77,78,79].

Despite the fact that several phytochemicals have exhibited significant chemopreventive activity in in vitro and in vivo models, their efficient translation to clinical studies, as single and/or combinatorial agents, has not provided substantial benefits due to several limitations, including low solubility, reduced stability, and low bioavailability [18,21]. For example, the chemopreventive efficacy of various agents is often reduced or even compromised in human studies because of the differences in their metabolism compared to both in vitro and in vivo pre-clinical models. Moreover, many agents are often converted into conjugated forms that may further account for their poor availability [80,81]. Another important aspect is the lack of optimized dosage along with time duration and mode of administration, all of which can result in non-physiological conditions [81,82]. Thus, the notion that the higher the agent’s dose, the better the beneficial/chemopreventive outcome, is unlikely to be realistic [83]. To this end, pharmacokinetic studies, improved bioavailability and better characterization of the underlying mode of action would enhance our understanding of the potential therapeutic efficacy of various agents (utilized in experimental therapeutic protocols either as single or combinatorial exposure platforms) and validate their potential to translate into the clinical setting.

## 5. Materials and Methods

### 5.1. Chemicals

RPMI-1640 and DMEM high glucose culture media, Fetal Bovine Serum (FBS), Phosphate Buffer Saline (PBS), L-glutamine and penicillin/streptomycin were purchased from Biosera (Kansas, MO, USA). Zebularine and SYBR green qPCR master mix (high rox) were purchased from MedChem (Stockholm, Sweden). SFN and IBN were purchased from Abcam (Cambridge, UK), while BITC was purchased from Sigma-Aldrich (St. Louis, MO, USA). Resazurin sodium salt was purchased from Fluorochem (Hadfield, UK). Caspases-3, -8 and -9 multiplex activity assay kit was obtained from Abcam (Cambridge, UK). PrimeScript^TM^ RT reagent kit was purchased from TaKaRa (Shiga, Japan) and NucleoZOL from Macherey-Nagel (Duren, Germany). Primers were purchased from Invitrogen (Waltham, MA, USA). Polyvinylidene difluoride (PVDF) membranes were purchased from Millipore (Bedford, MA, USA), whereas chemiluminescence reagents, proteinase, phosphatase inhibitors and the Pierce™ BCA protein assay kit were purchased from Thermo Scientific (Rockford, IL, USA). All antibodies (anti-DNMT1, anti-DNMT3A, anti-DNMT3B, anti-H3K4me2, anti-H3K4me3, anti-H3K9me2, anti-H3K9me3 and anti-H3K9ac) were obtained from Cell Signaling Technology (Boston, MA, USA), while anti-β actin was purchased from Sigma-Aldrich (Taufkirchen, Germany). Secondary horseradish peroxidase conjugated antibodies were purchased from Millipore (Bedford, MA, USA). Finally, glutaraldehyde (25%), osmium tetroxide, propylene oxide, araldite resin, agar 100 resin, dodenyl succinic anhydride (DDSA), 2,4,6-Tri(dimethylaminomethyl) phenol (DMP-30), uranyl acetate and lead citrate were all purchased from Agar Scientific (Essex, UK).

### 5.2. Cell Culture

A375 cells were purchased from American Type Culture Collection (ATCC; Cat No: CRL-1619, Manassas, VA, USA) whereas Colo-679 cells were purchased from DeutchSammlung von Mikroorganismen und Zellkultuten (DSMZ; Cat No: ACC 264, Braunscgweig, Germany). A375 and HaCaT cells were cultured in DMEM high glucose media, while Colo-679 were cultured in RPMI-1640 media. All culture media were supplemented with 10% FBS, 2 mM L-glutamine and 1% pen/strep (100 U/mL penicillin, 100 μg/mL streptomycin) and maintained in a humidified incubator at 37 °C and 5% CO_2_.

### 5.3. Exposure Protocols

Logarithmically growing A375, Colo-679 and HaCaT cells were seeded into 96-well culture plates (100 μL/mL) and incubated overnight at 37 °C and 5% CO_2_. The following day, the cells were exposed to increasing concentrations (35–100μM) of ZEB as a single agent over 24–72 h of exposure. Respective EC_50_ values were calculated using the CompuSyn software V.20 (Biosoft, Cambridge, UK). All experimental conditions were performed in pentaplicates, while experiments were repeated three independent times. In another set of experiments, A375 cells were seeded into 96-well plates (100 μL/wells at a density of 4000 cells/mL), and the following day, they were treated with ZEB and each of the ITCs over 72 h of exposure. Specifically, a range of different EC_50_ values of ZEB was combined with pre-determined EC_50_ values of SFN, IBN and BITC, as previously described [23,24]. To this end, combinatorial treatments of ZEB with each of the ITCs were evaluated as follows: (i) 100% of the EC_50_ of ZEB with 100% of the EC_50_ of each ITC, (ii) 75% of the EC_50_ of ZEB with 75% of the EC_50_ of each ITC, (iii) 50% of the EC_50_ of ZEB with 50% of the EC_50_ of each ITC and (iv) 25% of the EC_50_ of ZEB with 25% of the EC_50_ of each ITC (***Exposure Protocol 1***) (Figure S1). Next, A375 cells were exposed to combined treatments consisting of (i) gradually decreasing concentrations, by 2.5 μM, of the selected EC_50_ of ZEB together with (ii) gradually increasing concentrations, by 2.5 μM, of the EC_50_ of each ITC and up to 20 μΜ concentration, as previously described [23,24] (***Exposure Protocol 2***) (Figure S2). All combined concentrations of ZEB with each ITC were also used for exposing HaCaT cells (Table S1). Finally, the combinational concentrations that caused a decrease of around 50% in cell viability in A375 and Colo-679 cells but maintained viability over 85% in HaCaT cells were selected as optimized exposure conditions in all subsequent experiments described thereafter. Specifically, our optimized combinational protocol consisted of the following experimental conditions: (i) 35 μΜ of ZEB + 10 μM SFN, (ii) 30 μΜ ZEB + 15 μM IBN and (iii) 35 μΜ ZEB + 10 μM BITC (Table S1).

### 5.4. Determination of Cell Viability

A total of 8000, 4000 and 2000 A375 cells/well or 10000, 5000 and 2500 Colo-679 or HaCaT cells/well were seeded for 24, 48 and 72 h of exposure. The following day, cells were exposed to either ZEB alone or in combination with either SFN, IBN or BITC. The determination of cell viability was performed by the Alamar Blue assay and was expressed as a percentage of control (untreated) cells.

### 5.5. Combination Index Analyses

Combination index analyses of ZEB with each of the ITCs were performed using the CompuSyn software, V.20 (Biosoft, Cambridge, UK) (Figure S3). According to the Chou-Talalay method, the interactions between ZEB and each ITC were determined by calculating the Combination Index (CI), which is considered the standard measure of any combinatorial effect. Specifically, the analysis with CompuSyn software revealed CI values [over fractional cell death levels (Fa)] ranging from 0.05 to 0.95. Overall, values of CI < 1, CI = 1, and CI > 1 suggest either a synergistic, additive or antagonistic effect, respectively [84,85]. Specifically, a synergistic effect is observed when the combined effect of two compounds is greater than the sum of the effects of each compound being administrated alone. An antagonistic effect is observed when the combination of two compounds causes a decrease in the effect of one or both of them. Finally, an additive effect is observed when the combinatorial effect of both compounds equals the sum of the effects of each compound when acting independently [86] (Table S2).

### 5.6. Determination of Caspase-3 Activity Levels

A375 and Colo-679 cells were grown at a density of 4000 and 5000 cells/well in black 96-well plates, respectively, before being exposed to any experimental condition for a total period of 72 h. Measurements were performed by utilizing the Caspases-3, -8 and -9 Multiplex Activity Assay Kit. Briefly, a caspase loading solution consisting of 50 μL of caspase-3 substrate was mixed with 10 mL of assay buffer, and subsequently, 100 μL of caspase loading solution was added to each well without the removal of culture media. Afterwards, cells were left at room temperature (RT), in the dark, for 1 h, while the fluorescence intensity was measured using a fluorescence microplate reader (Synergy H1, Bio-Tek, Shoreline, WA, USA) at 535/620 nm excitation/emission wavelengths. Finally, blank-corrected values (Relative Fluorescence Units; RFUs) related to each treatment group were represented as fold change from the negative control (untreated samples).

### 5.7. Real-Time Polymerase Chain Reaction (RT-PCR)

A375 and Colo-679 cells were seeded at a density of 1 × 10^6^ and 1.2 × 10^6^ cells, respectively, in 100 mm petri dishes. After exposures, the cells were trypsinized and collected, and total NucleoZOL Reagent was used for RNA extraction. RNA quality was determined using the NanoDrop One/OneC (Thermo Scientific, Waltham, MA, USA). Complementary DNA (cDNA) was synthesized using the PrimeScript™ First Strand cDNA Synthesis kit (TaKaRa, Dalian, China). RT-PCR was performed using a StepOne Plus RT-PCR instrument (Applied Biosystems, Carlsbad, CA, USA) using SYBR Green. The sequences of the primers used are provided in Table S3. Gene expression data were normalized to GAPDH using the 2-ΔΔCt method and were expressed as fold-change compared to untreated (control) samples.

### 5.8. Western Immunoblotting

Following exposures, A375 cells were collected, washed in PBS, lysed with RIPA lysis buffer (10 mM Tris-HCl, pH 8.0; 1 mM EDTA; 0.5 mM EGTA; 1% Triton X-100; 0.1% Sodium Deoxycholate;0.1% SDS; 140 mM NaCl) and supplemented with a cocktail of proteinase and phosphatase inhibitors. Lysates were incubated for 30 min at 4 °C, vortexed every 10 min and centrifuged at 14000× *g* for 15 min to prepare whole-cell extracts. The determination of protein concentration was performed by utilizing the Pierce™ BCA Protein Assay kit. Afterwards, whole cell extracts (50 µg) were prepared and subjected to SDS-PAGE on 8%, 10% and 12% Tris-Glycine gels. Separated proteins were transferred to PVDF membranes (0.2 and/or 0.45 mm) using the Trans-Blot Turbo Transfer System (BioRad, Hercules, CA, USA) while non-specific sites were blocked with 5% non-fat dry milk in 150 mM NaCl, 100 mM Tris pH 7.5 and 0.1% (*v*/*v*) Tween-20 at room temperature for 2 h. Next, the membranes were hybridized overnight at 4 °C with primary antibody at different dilutions against anti-DNMT1 (1/1000), anti-DNMT3A (1/1000), anti-DNMT3B (1/2000), anti-H3K4me2 (1/1000), anti-H3K4me3 (1/1000), anti-H3K9me2 (1/1000), anti-H3K9me3 (1/1000), anti-H3K9ac (1/1000) and anti-β-actin (1/20000). Then, the membranes were incubated with appropriate secondary horseradish peroxidase conjugated IgG antibodies (1/1000) for 1 h at RT, while protein bands were developed using the SuperSignal West Pico PLUS Chemiluminescent Substrate. All images were obtained with a BioSpectrum 810 imaging system (UVP) while blots were stripped with a stripping buffer (62.5 mM Tris-HCl pH 6.7, 2% SDS, 6 µL/mL 2-mercaptoethanol) and re-probed with an appropriate antibody. For the detection of equal protein loading, each membrane was stripped and re-probed with anti β-actin antibody.

### 5.9. Transmission Electron Microscope (TEM) Imaging

A375 cells were treated with either ZEB (30 μΜ/35 μΜ) or each ITC alone (10 μM SFN, 15 μM IBN, 10 μΜ BITC) for 72 h. In combined exposures, A375 cells were pre-treated with ZEB for 24 h, followed by the addition of either SFN (35 μΜ ZEB/10 μΜ SFN), IBN (30 μΜ ZEB/15 μΜ IBN) or BITC (35 μΜ ZEB/10 μΜ BITC) for an additional 48 h (72 h total). Following exposures, trypsinized cells were collected and washed twice with ice-cold PBS. The obtained pellets were fixed in 2.5% glutaraldehyde (0.1 M phosphate buffer, pH 7.2) for at least 24 h at 4 °C. Then, fixed cells were thoroughly washed with phosphate buffer (0.1 M, pH 7.2) before being post-fixed with 1% osmium tetroxide. Afterwards, fixed samples were dehydrated with a series of graded ethanol (50–100%), cleared in propylene oxide and embedded in an epon/araldite resin mixture. The polymerization occurred at 60 °C for 24 h. Semithin sections of 1 μm thickness were cut with an ultra-microtome (Leica, Reichert UCT, Vienna, Austria), stained with toluidine blue and observed under a light microscope. Gold interference color ultrathin sections (70 nm thickness) were then cut and mounted with 300 mesh copper grids contrasted with uranyl acetate-zero and lead citrate. The stained grids were examined under a TEM (TALOS L120C, Thermo Scientific, Waltham, MA, USA) operating at 120 kV, using a CETA camera.

### 5.10. Statistical Analysis

Data were expressed as mean values ± standard error of the mean (SEM), and comparisons were performed between untreated (control) and treated samples. Statistical analyses were performed by one-way analysis of variance (one way-ANOVA) with Tukey’s test for multiple comparisons, using Graph Pad Prism (GraphPad software 10.4.1, San Diego, CA, USA). Values of *p* < 0.05, *p* < 0.01 and *p* < 0.001 were considered statistically significant compared to untreated (control) samples.

## Figures and Tables

**Table 1:** Half-maximal response (EC_50_) values of ZEB against human malignant melanoma (A375, Colo-679) cells. Cells were exposed to increasing concentrations of ZEB (35–100 μM) for 24–72 h, and EC_50_ values were calculated using the CompuSyn software, V.20 (Biosoft, Cambridge, UK). N.D: Not Determined.

Cell Line	Exposure Period (h)	EC_50_ (μΜ)
A375	24	N.D
	48	75.56
	72	48.42
Colo-679	24	N.D
	48	84.17
	72	56.45
	24	N.D
HaCaT	48	N.D
	72	N.D

## Data Availability

All data are available within this manuscript, including Supplementary Material.

## Supplementary Materials

The following supporting information can be downloaded at: https://www.mdpi.com/article/10.3390/epigenomes9010007/s1, Figure S1: Cell viability levels of malignant melanoma cells following Exposure Protocol 1; Figure S2: Cell viability levels of malignant melanoma cells following Exposure Protocol 2; Figure S3: Combination Index (CI) plot of combinatorial exposures between ZEB and each ITC in human malignant melanoma cells; Table S1: Cell viability levels of A375 and HaCaT cells following various co-exposure conditions between different concentrations of ZEB with each ITC; Table S2: Interaction analysis between co-exposures of ZEB with each ITC in A375 cells; Table S3: List of primer sequences used in RT-PCR experiments; Table S4: Fold change in expression levels of apoptotic genes in A375 cells exposed to either ZEB alone or in combination with each ITC; Table S5: Fold change in expression levels of apoptotic genes in Colo-679 cells exposed to either ZEB alone or in combination with each ITC.
