# Supplementary material for: Isothiocyanates Enhance the Anti-Melanoma Effect of Zebularine Through Modulation of Apoptosis and Regulation of DNMTs’ Expression, Chromatin Configuration and Histone Posttranslational Modifications Associated with Altered Gene Expression Patterns
Source: Epigenomes. 2025 Feb 25;9(1):7. doi: 10.3390/epigenomes9010007 (PMC11941220; doi:10.3390/epigenomes9010007)
Supplement: Supplementary file 1 [file epigenomes-09-00007-s001.zip › epigenomes-3448047-supplementary.pdf]

SUPPLEMENTARY DATA

Isothiocyanates enhance the anti-melanoma effect of Zebularine through modulation of apoptosis and regulation of DNMTs' expression, chromatin configuration and histone posttranslational modifications associated with altered gene expression patterns

Ioannis Anestopoulos[1#], Ioannis Paraskevaidis[2#], Sotiris Kyriakou[1], Louiza Potamiti[1], Dimitrios T. Trafalis[3], Sotiris Botaitis[4], Rodrigo Franco[5,6], Aglaia Pappa[7], Mihalis I. Panayiotidis[1,8]*

[#]co-first authors

[1]Department of Cancer Genetics, Therapeutics & Ultrastructural Pathology, The Cyprus Institute of Neurology & Genetics, Nicosia 2371, Cyprus; [2]Perelman School of Medicine, University of Pennsylvania, Philadelphia, PA19104, USA; [3]Laboratory of Pharmacology, Medical School, National & Kapodistrian University of Athens, Athens 11527, Greece; [4]Department of Surgery, University Hospital, Democritus University of Thrace, School of Medicine, Alexandroupolis 68100, Greece; [5]School of Veterinary Medicine & Biomedical Sciences, University of Nebraska-Lincoln, Lincoln, NE 68583, USA; [6]Redox Biology Centre, University of Nebraska-Lincoln, Lincoln, NE 68583, USA; [7]Department of Molecular Biology & Genetics, Democritus University of Thrace, Alexandroupolis 68100, Greece; [8]Department of Comparative Biomedical Sciences, School of Veterinary Medicine, Mississippi State University, Starkville, MS 39762, USA

*Corresponding author: Prof. Dr. Mihalis I. Panayiotidis; Department of Cancer Genetics, Therapeutics & Ultrastructural Pathology, The Cyprus Institute of Neurology & Genetics, 6

Iroon Avenue, Ayios Dometios, Nicosia 2371, Cyprus. E-mail: mihalisp@cing.ac.cy; and Department of Comparative Biomedical Sciences, College of Veterinary Medicine, Mississippi State University, P.O. Box 6100, 240 Wise Center Dr., Starkville, MS 39762, USA. E-mail: mp2358@msstate.edu

Figure S1

S1A.

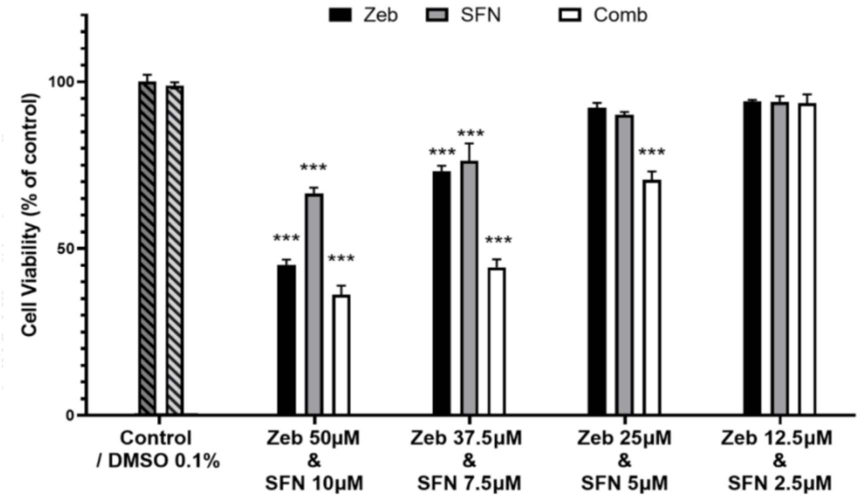

S1B.

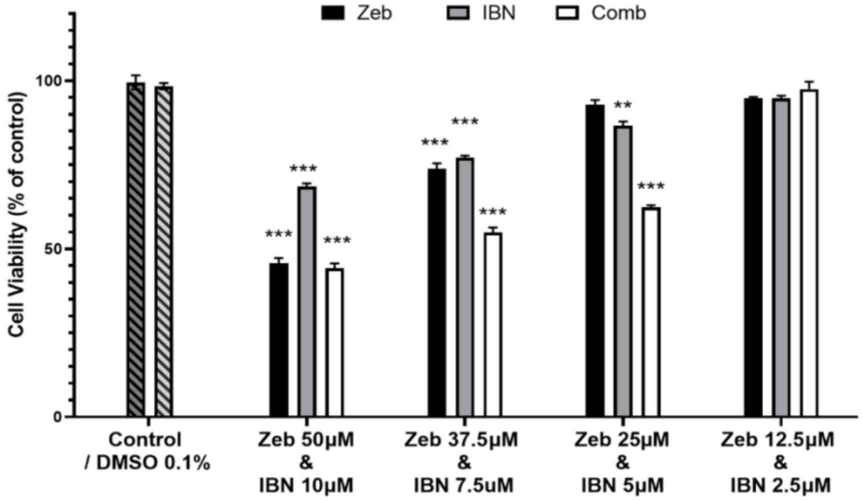

S1C.

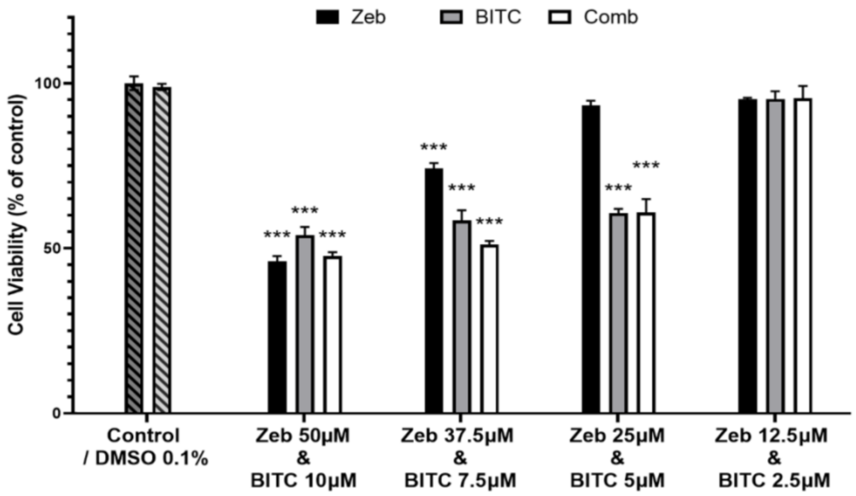

Fig. S1: Cell viability levels of human melanoma cells following Exposure Protocol 1. A375cells were exposed either to ZEB or each ITC [e.g., SFN **(S1A)**, IBN **(S1B)** and BITC **(S1C)**] alone or in combinatorial exposures between ZEB with each ITC as follows: i) 100% of the EC_{50} of ZEB (50μM) with 100% of the EC_{50} of each ITC (10μM),ii) 75% of the EC_{50} of ZEB (37.5μM) with 75% of the EC_{50} of each ITC (7.5μM), iii) 50% of the EC_{50} of ZEB (25μM) with 50% of the EC_{50} of each ITC (5μM) and iv) 25% of the $EC_{50}$of ZEB(12.5μM) with 25% of the EC_{50} of each ITC (2.5μM). All data are expressed as means ± SEM and are representative of three independent experiments. Statistical significance is indicated by *$p < 0.05$, **$p<0.01$ and ***$p<0.001$ relative to the corresponding 0.1% DMSO controls.

<u>Figure S2</u>

S2A.

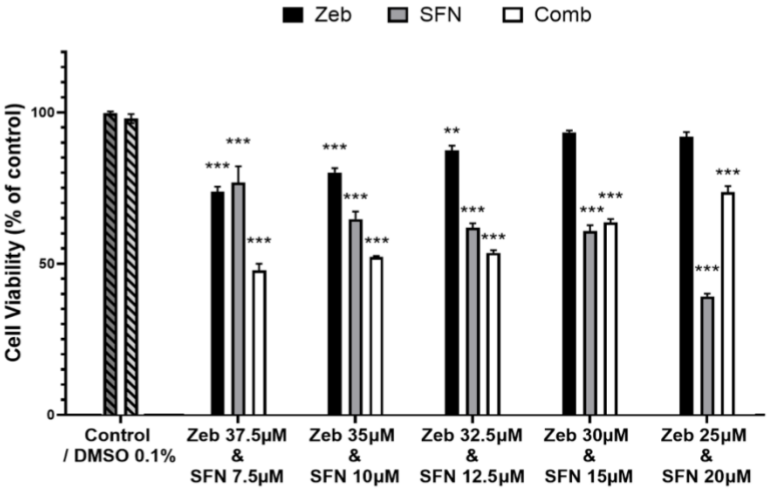

S2B.

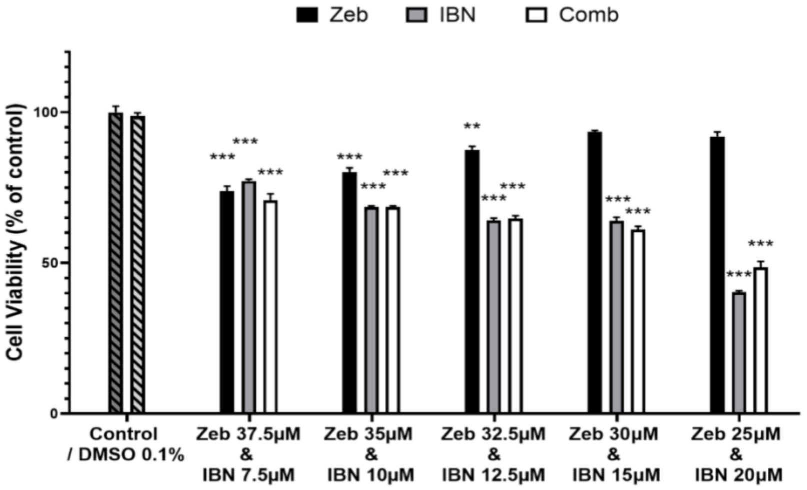

S2C.

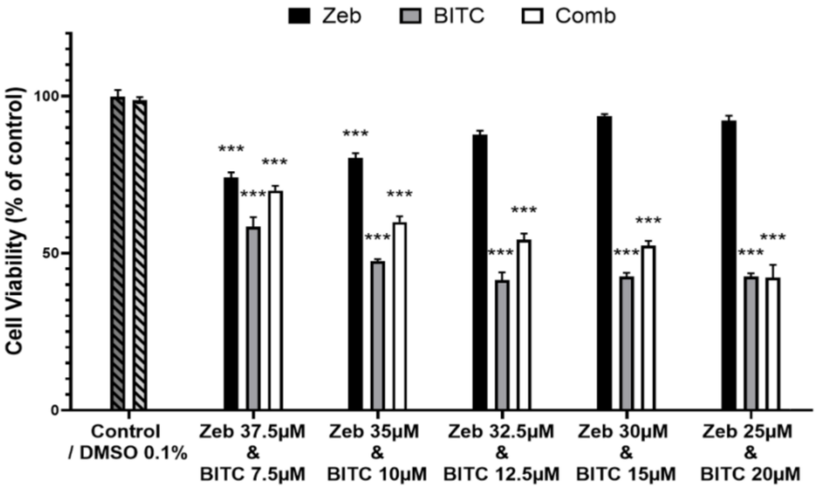

Fig.S2:Cell viability levels of malignant melanoma cells following Exposure Protocol2.

A375 cells were exposed either to ZEB or each ITC [e.g., SFN **(S2A)**, IBN **(S2B)** and BITC

(S2C)] alone or in combinatorial exposures with ZEB and each ITC by gradually decreasing the EC_{50} of ZEB by 2.5μM with a parallel gradual increase of the EC_{50} of each ITC by 2.5μM until reached a final concentration of 20μM. All data are expressed as means ± SEM and are representative of three independent experiments. Statistical significance is indicated by *p < 0.05, **p<0.01 and ***p<0.001 relative to the corresponding 0.1% DMSO controls.

Figure S3

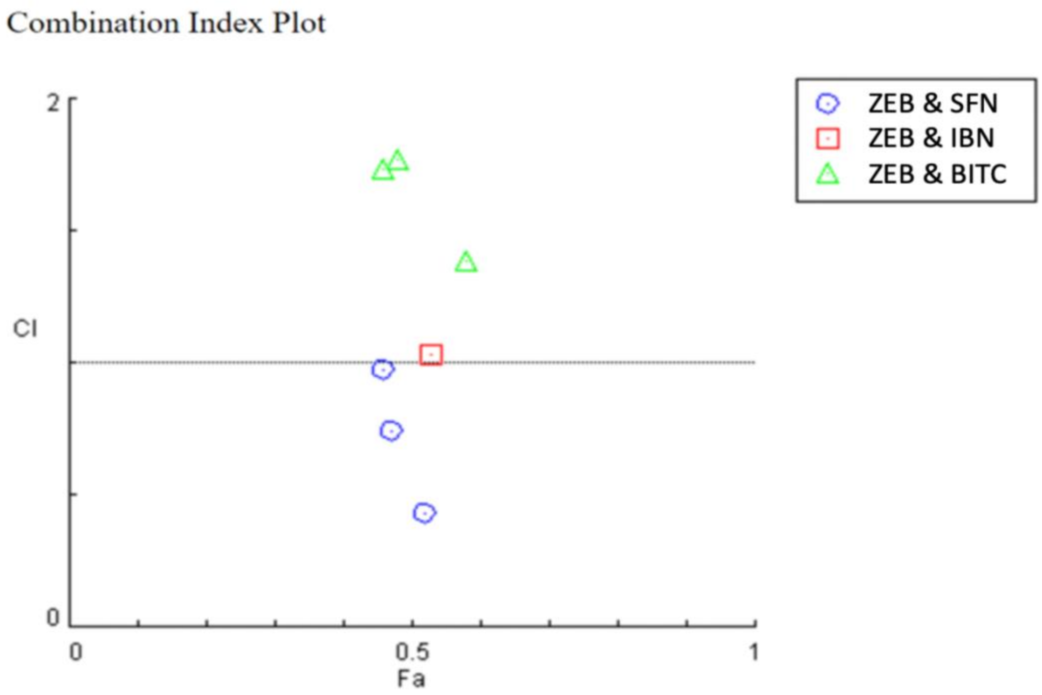

Fig.S3: Combination Index (CI) plot of combinatorial exposures between ZEB and each ITC in human malignant melanoma cells. Combination Index (CI) plot descriptive of the combinatorial exposures between ZEB with SFN, IBN and BITC in A375 cells. The type of interaction of each combined treatment was determined through the CompuSyn software, V.20 (Biosoft, Cambridge, UK) and were interpreted either assynergistic (CI<1), additive (CI=1) or antagonistic (CI>1).

Table S1: Cell viability levels of A375 and HaCaT cells following various co-exposure conditions between different concentrations of ZEB with each ITC. The rationale for the indicated co-exposure conditions was based on their efficacy to sustain cytotoxicity in A375 cells approximately to their EC_{50} value (50-60%) while at the same time maintain viability to neighboring non-malignant keratinocyte (HaCaT) cells close to control levels (90% and above). In this context, bold highlighted exposure conditions were selected as optimumand wereutilized in all experiments thereafter. Data are expressed as means ± SEM and are representative of three independent experiments.

Exposure Protocol	A375	HaCaT
Control	100.00 ±3.09	100.00 ± 2.04
0.1% DMSO	111.61±2.50	111.61±2.50
Zeb (50.0μM)	50.58±1.38	103.11±2.98
Zeb (37.0μM)- SFN (7.5μM)	48.18±2.19	99.60±2.15
**Zeb (35.0μM)- SFN (10.0μM)**	**52.61±0.36**	**102.68±1.83**
Zeb (32.5μM)- SFN (12.5μM)	53.97±0.92	62.10±3.21
**Zeb (30.0μM)- IBN (15.0μM)**	**61.49±1.10**	**95.59±4.12**
Zeb (25.0μM)- IBN (20.0μM)	48.92±1.95	60.38±4.19
**Zeb (35.0μM)- BITC (10.0μM)**	**60.04±1.81**	**88.32±3.82**
Zeb (32.5μM)- BITC (12.5μM)	54.38±1.99	80.06±1.75
Zeb (30.0μM)- BITC (15.0μM)	52.47±1.58	70.93±1.78

Table S2: Interaction analysis between co-exposures of ZEBwith each ITC in A375cells. Analysis was based on the Chou-Talalay technique where all potential interactions were identified by calculating the Combination Index (CI) using CompuSyn software, V.20 (Biosoft, Cambridge, UK). All possible interactions were interpreted either assynergistic (CI<1), additive (CI=1) or antagonistic (CI>1)

TREATMENT	COMBINATION INDEX (CI)	INTERACTION
ZEB (35μM) + SFN (10μM)	0.74	Synergistic
ZEB (30μM) + IBN (15μM)	1.11	Additive
ZEB (35μM) + BITC (10μM)	4.94	Antagonistic

Table S3: List of primer sequences used in RT-PCR experiments

GENE	FORWARD PRIMER	REVERSE PRIMER
**INTRINSIC APOPTOTIC PATHWAY**		
CASP2	5’- ATTGGATCCCTTGGGCACCTC-3’	5’ ATCATGTCTGAGCGCGTGGG-3’
CASP3	5’ TTATTCAGGCCTGCCGTGGT-3’	5’- AGCATGGCACAAAGCGACTG-3’
CASP6	5- TTTGGCTGCAATGAGCTCGG-3’	5’- GGCATCTGCGTGGCTAACAG-3’
CASP7	5’- AAGCTGAGGGAGCGTCCTAC-3’	5- ACCGGTCTGGCTTAGCATCC-3’
CASP9	5’-CGGTGACGCAAGAGCGAATC-3’	5- GATCAGCTGCCTGGCCTGAT-3’
APAF1	5’- GTTGGGTTCATGGTGTGATG-3’	5’- TTTGTCTCCCAGAGCCTGA-3’
BAD	5’- TCAGGGGCCTCGAGATCGG-3’	5’- TCCTGCTCACTCGGCTCAAA-3’
BAK1	5’- AGACCTGAAAAATGGCTTCG	5’- CGGAAAACCTCCTCTGTGTC-3’
BCL2	5’- AGTACCTGAACCGGCACCT-3’	5’- GCCGTACAGTTCCACAAAGG-3’
BID	5’- TGCAGCTCAGGAACACCA-3’	5’-TCTCCATGTCTCTAGGGTAGGC-3’
BAX	5’- ACCTTGACTTGATTAGTGCCTTCT-3’	5’- GGGTCATCAATGAACTTGAGC-3’
XIAP	5’- GCAAGAGCTCAAGGAGACCA-3’	5- AAGGGTATTAGGATGGGAGTTCA-3’
MCL1	5’- TTACGACGGGTTGGGGATGG-3’	5’- CTGCCCCAGTTTGTTACGCC-3’
CYCS	5’- TTCGGAGCGGGAGTGTTCGT-3’	5’- TGTGGCACTGGGAACACTTCAT-3’
DIABLO	5’- TGACTGCAGTTGGTCTTTCAG-3’	5’- GCGGTTATAGAGGCCTGATCT-3’
BCL2L11	5’- ACTGGAGAGCTCATTGCAGAC-3’	5’- AAATACCAGGACCCGAAGGT-3’
BCL2L1	5’- CGTGGAAAGCGTAGACAAG-3’	5’- AAGAGTGAGCCCAGCAGAA-3’
PMAIP1	5’- CAAGAACGCTCAACCGAGCC-3’	5’- AGGAGTCCCCTCATGCAAGT-3’
**EXTRINSIC APOPTOTIC PATHWAY**		
CASP8	5’- GGCGGAGGGTCGATCATCTAT-3’	5’- TTCCTTCTCCCAGGATGACCC-3’
CASP10	5’- GGTAGTAATGAGGGCAGCTGTGT-3’	5’- TGAACCCAAGCCACTGGAACA-3’
FAS	5’- TTTTCCTCATGGCTTCACCT-3’	5’- ATGTGGCTGTGCTCATTGAC-3’
BIRC2	5’- ATCGTGCGTCAGAGTGAGC-3’	5’- CGCCGACAAGGAGATACG-3’
BIRC3	5’- GCTTGTCCTTGCTGGTGCAT-3’	5’- TCCCGAGATTAGACTAAGTCCCTT-3’
C-FLAR	5’- TCCATCTTGGGTGCGCCTTC-3’	5’- TCCGGGCCAGTCAACAGAAA-3’
FADD	5’- CCGAGCTCAAGTTCCTATGC-3’	5’- AGGTCTAGGCCGCTCTGC-3’
FAIM2	5’- ATCGGAGCGAAGCAGAGAGG-3’	5’- CACAGTGGTTGAGCATGGGC-3’
FASLG	5’- GAGTCTACCAGCCAGATGCACA-3’	5’- AGGCATGGACCTTGAGTTGG-3’
TNFRSF10A	5’- TACGCCCTGGAGTGACATCG-3’	5’- GACCCAAGCGCCAGAAACAC-3’
TNFRSF10C	5’- CCCTAAAGTTCGTCGTCGTC-3’	5’- TGGTGGCAGAGTAAGCTAGGA-3’
TNF	5’- TGCACTTTGGAGTGATCGGC-3’	5’- TTGTCACTCGGGGTTCGAGA-3’
TNFAIP3	5’- TGCACACTGTGTTTCATCGAG-3’	5’- ACGCTGTGGGACTGACTTTC-3’
TNFRSF10D	5’- AAGTTCGTCGTCTTCATCGTC-3’	5’- GATGGTGGCAGAGTCAACC-3’
TNFRSF1B	5’- CCAGTGCGTTGGACAGAAGG-3’	5’- ATGGCCACCAGGGGAAGAAT-3’
TNFRSF10	5’- TCACAGTGCTCCTGCAGTCT-3’	5’- GCCACTTTTGGAGTACTTGTCC-3’
TRADD	5’- TAGTGCAGCAGGAGGTGAGATG-3’	5’- CCAGCGAGGACTCCACAAAC-3’
TRAF2	5’- GCATACCCGCCATCTTCTC-3’	5’- CGCCGTTCAGGTAGATACG-3’
TRAF5	5’- ATGCCACTTCCCTACTGCTC-3’	5’- CATGGCCCATACTCATCCTT-3’
GAPDH	5’-GACAGTCAGCCGCATCTTCT-3’	5’-GCGCCCAATACGACCAAATC - 3’

	ZEB	ZEB + SFN	ZEB + IBN	ZEB + BITC
CASP3	**1.827**± 0.317	**2.293**± 0.405	**2.159**± 0.211	**2.342**± 0.008
BID	**2.250**± 0.054	**2.532**± 0.389	**1.672**± 0.036	**1.177**± 0.06
BAX	**1.669**± 0.303	**5.012**± 0.041	**3.750**± 0.004	**2.336**± 0.174
XIAP	**0.725**± 0.113	**0.840**± 0.037	**1.202**± 0.107	**0.769**± 0.063
PMAIP1	**1.425**± 0.107	**1.597**± 0.056	**0.743**± 0.075	**1.937**± 0.003
BAD	**1.337**± 0.021	**1.191**± 0.402	**1.175**± 0.024	**1.279**± 0.066
CYCS	**2.195**± 0.045	**5.018**± 0.007	**1.508**± 0.027	**4.563**± 0.006
BAK1	**1.526**± 0.028	**1.492**± 0.003	**1.682**± 0.023	**2.385**± 0.006
APAF1	**1.041**± 0.969	**1.451**± 0.782	**1.151**± 0.006	**1.025**± 0.332
CASP9	**1.096**± 0.029	**1.001**± 0.229	**1.131**± 0.124	**1.065**± 0.122
CASP2	**1.052**± 0.020	**1.163**± 0.039	**0.767**± 0.075	**0.979**± 0.671
DIABLO	**0.797**± 0.011	**0.777**± 0.010	**0.847**± 0.118	**1.310**± 0.003
BCL2L11	**1.830**± 0.247	**1.040**± 0.482	**1.435**± 0.001	**1.622**± 0.136
CASP6	**1.655**± 0.042	**2.584**± 0.062	**2.984**± 0.062	**1.784**± 0.121
CASP7	**1.586**± 0.069	**1.228**± 0.077	**1.126**± 0.196	**1.132**± 0.076
MCL1	**0.508**± 0.067	**0.582**± 0.019	**0.534**± 0.002	**0.394**± 0.072
BCL2	**0.312**± 0.017	**0.003**± 0.336	**0.084**± 0.109	**0.406**± 0.016
BCL2L1	**1.131**± 0.053	**1.163**± 0.133	**0.770**± 0.030	**1.156**± 0.010

FASL	**1.291**± 0.329	**1.023**±0.032	**1.203**± 0.023	**1.420**± 0.932
FAS	**1.182**± 0.046	**1.105**±0.168	**1.842**± 0.152	**1.288**± 0.021
FAIM2	**1.188**± 0.269	**1.024**±0.365	**1.621**± 0.150	**1.039**± 0.136
TNFRSF10	**1.011**± 0.044	**1.096**±0.287	**1.043**± 0.886	**1.092**± 0.453
TNFRSF10D	**0.379**± 0.059	**0.119**±0.891	**0.011**± 0.858	**0.028**± 0.072
TNFRSF10C	**0.406**± 0.029	**0.302**±0.681	**0.325**± 0.672	**0.369**± 0.521
TNFRSF10B	**2.110**± 0.043	**5.192**±0.227	**6.013**± 0.605	**4.816**± 0.032
TNFRSF10A	**2.344**± 0.943	**5.823**±0.341	**3.126**± 0.070	**2.851**± 0.109
FADD	**1.192**± 0.855	**2.569**±0.065	**3.316**± 0.582	**2.563**± 0.123
CASPASE8	**1.935**± 0.027	**4.006**±0.395	**3.009**± 0.031	**3.011**± 0.096
CASPASE10	**1.023**± 0.032	**1.052**±0.039	**1.009**± 0.320	**1.420**± 0.242
C-FLAR	**0.893**± 0.007	**0.675**±0.046	**0.715**± 0.057	**0.646**± 0.007
TNF	**1.937**± 0.005	**1.001**±0.076	**0.924**± 0.260	**0.977**± 0.077
TNFRSF1A	**1.167**± 0.154	**1.749**±0.031	**1.891**± 0.030	**1.476**± 0.044
TRADD	**0.274**± 0.102	**0.170**±0.938	**0.522**± 0.796	**0.459**± 0.233
TRAF2	**0.792**± 0.131	**0.911**±0.032	**0.817**± 0.030	**0.617**± 0.022
BIRC2	**0.556**± 0.029	**0.586**±0.246	**0.649**± 0.837	**0.608**± 0.155
BIRC3	**0.632**± 0.021	**0.521**±0.174	**0.737**± 0.323	**0.644**± 0.104
TRAF1	**0.982**± 0.130	**1.203**±0.532	**0.942**± 0.420	**1.320**± 0.123
TRAF5	**1.062**± 0.092	**1.076**±0.258	**1.013**± 0.292	**1.087**± 0.486
TNFAIP3/A20	**0.810**± 0.029	**1.170**±0.417	**0.850**± 0.213	**0.762**± 0.151

Table S4: Fold change in expression levels of apoptotic genesin A375 cells exposedto either ZEB alone or in combination with each ITC. Specifically, A375 cells were pre-exposed, for 24h,to ZEB alone followed by the addition of each ITC for further 48h and for a total of 72h of exposure. Fold changes in gene expression levels were determined by Real-Time PCR. All data are expressed as means of three replicates ± SEM and are representative of three independent experiments. Gene expression data were normalized to GAPDH using the $2^{-\Delta\Delta Ct}$ method and were expressed as fold-change compared to untreated (control) samples

	ZEB	ZEB + SFN	ZEB + IBN	ZEB + BITC
CASP3	**1.431**± 0.033	**2.492**± 0.104	**2.293**± 0.029	**2.882**± 0.102
BID	**1.105**± 0.029	**1.922**± 0.029	**1.827**± 0.039	**1.210**± 0.082
BAX	**1.702**± 0.102	**3.102**± 0.029	**2.918**± 0.192	**2.001**± 0.293
XIAP	**1.029**± 0.029	**0.910**± 0.020	**0.829**± 0.210	**1.211**± 0.029
PMAIP1	**1.219**± 0.029	**1.420**± 0.039	**1.210**± 0.239	**1.529**± 0.039
BAD	**1.102**± 0.020	**1.201**± 0.210	**1.320**± 0.042	**1.429**± 0.083
CYCS	**1.902**± 0.039	**4.203**± 0.193	**2.102**± 0.039	**3.909**± 0.102
BAK1	**1.422**± 0.012	**1.209**± 0.020	**1.210**± 0.389	**1.792**± 0.329
APAF1	**1.210**± 0.093	**1.019**± 0.214	**1.001**± 0.291	**1.290**± 0.320
CASP9	**1.929**± 0.029	**1.201**± 0.089	**0.902**± 0.102	**1.429**± 0.139
CASP2	**0.920**± 0.019	**1.320**± 0.021	**0.981**± 0.028	**1.210**± 0.098
DIABLO	**1.102**± 0.012	**0.929**± 0.019	**1.102**± 0.019	**1.029**± 0.032
BCL2L11	**1.602**± 0.109	**1.290**± 0.019	**1.298**± 0.097	**1.249**± 0.047
CASP6	**1.201**± 0.039	**1.982**± 0.039	**1.748**± 0.039	**1.498**± 0.029
CASP7	**1.320**± 0.209	**1.429**± 0.029	**1.821**± 0.201	**1.392**± 0.039
MCL1	**0.723**± 0.039	**0.892**± 0.492	**0.728**± 0.291	**0.672**± 0.019
BCL2	**0.879**± 0.029	**0.728**± 0.293	**0.592**± 0.029	**0.821**± 0.029
BCL2L1	**0.929**± 0.019	**1.019**± 0.012	**1.299**± 0.021	**1.921**± 0.094

FASL	**1.029**± 0.353	**1.219**± 0.429	**1.001**± 0.038	**0.982**± 0.129
FAS	**1.423**± 0.042	**1.527**± 0.429	**1.927**± 0.029	**1.492**± 0.053
FAIM2	**1.392**± 0.029	**1.392**± 0.013	**1.428**± 0.046	**1.193**± 0.054
TNFRSF10	**1.203**± 0.044	**1.329**± 0.048	**1.562**± 0.047	**1.369**± 0.039
TNFRSF10D	**0.520**± 0.091	**0.232**± 0.052	**0.102**± 0.013	**0.332**± 0.024
TNFRSF10C	**0.492**± 0.039	**0.301**± 0.064	**0.293**± 0.043	**0.293**± 0.023
TNFRSF10B	**1.992**± 0.049	**3.499**± 0.047	**4.291**± 0.206	**4.204**± 0.032
TNFRSF10A	**2.019**± 0.023	**4.392**± 0.291	**2.992**± 0.392	**2.910**± 0.078
FADD	**1.521**± 0.021	**2.501**± 0.043	**2.482**± 0.021	**2.192**± 0.039
CASPASE8	**1.994**± 0.103	**3.209**± 0.049	**3.199**± 0.092	**2.729**± 0.057
CASPASE10	**1.209**± 0.047	**1.384**± 0.058	**1.329**± 0.047	**1.492**± 0.019
C-FLAR	**1.019**± 0.042	**0.902**± 0.019	**0.991**± 0.210	**0.028**± 0.042
TNF	**1.429**± 0.019	**1.291**± 0.027	**1.219**± 0.028	**1.019**± 0.056
TNFRSF1A	**1.102**± 0.309	**1.428**± 0.047	**1.682**± 0.044	**1.582**± 0.039
TRADD	**0.931**± 0.029	**0.829**± 0.013	**0.758**± 0.219	**0.821**± 0.121
TRAF2	**0.739**± 0.056	**0.924**± 0.049	**0.703**± 0.016	**0.721**± 0.012
BIRC2	**0.601**± 0.047	**0.712**± 0.024	**0.593**± 0.109	**0.791**± 0.059
BIRC3	**0.570**± 0.101	**0.839**± 0.025	**0.724**± 0.219	**0.619**± 0.214
TRAF1	**1.211**± 0.391	**1.029**± 0.112	**1.192**± 0.022	**1.472**± 0.024
TRAF5	**1.213**± 0.129	**1.194**± 0.019	**1.221**± 0.012	**1.329**± 0.583
TNFAIP3 / A20	**1.120**± 0.311	**1.112**± 0.028	**0.914**± 0.056	**0.938**± 0.427

Table S5:Fold change in expression levels of apoptotic genes in Colo-679 cells exposed to either ZEB alone or in combination with each ITC. Specifically, Colo-679 cells were pre-exposed, for 24h,to ZEB alone followed by the addition of each ITC for further 48h and for a total of 72h of exposure. Fold changes in gene expression levels were determined by Real-Time PCR. All data are expressed as means of three replicates ± SEM and are representative of three independent experiments. Gene expression data were normalized to GAPDH using the $2^{-\Delta\Delta Ct}$ method and were expressed as fold-change compared to untreated (control) samples

Un-cropped western immunoblot membranes in Figure 4

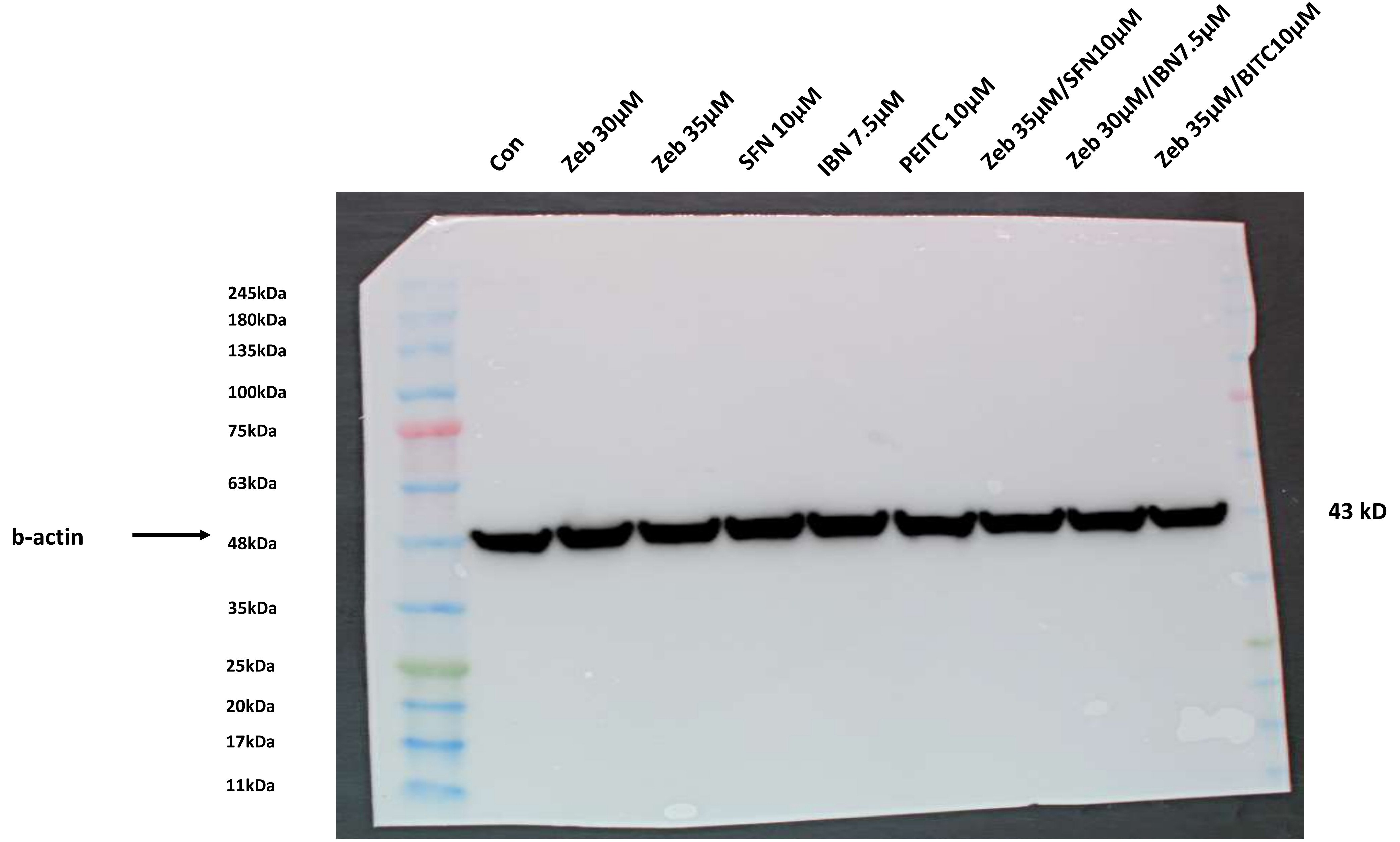

Con
Zeb 30µM
Zeb 35µM
SFN 10µM
IBN 7.5µM
PEITC 10µM
Zeb 35µM/SFN10µM
Zeb 30µM/IBN7.5µM
Zeb 35µM/BITC10µM
245kDa
180kDa
135kDa
100kDa
75kDa
63kDa
b-actin
48kDa
35kDa
25kDa
20kDa
17kDa
11kDa
43 kD

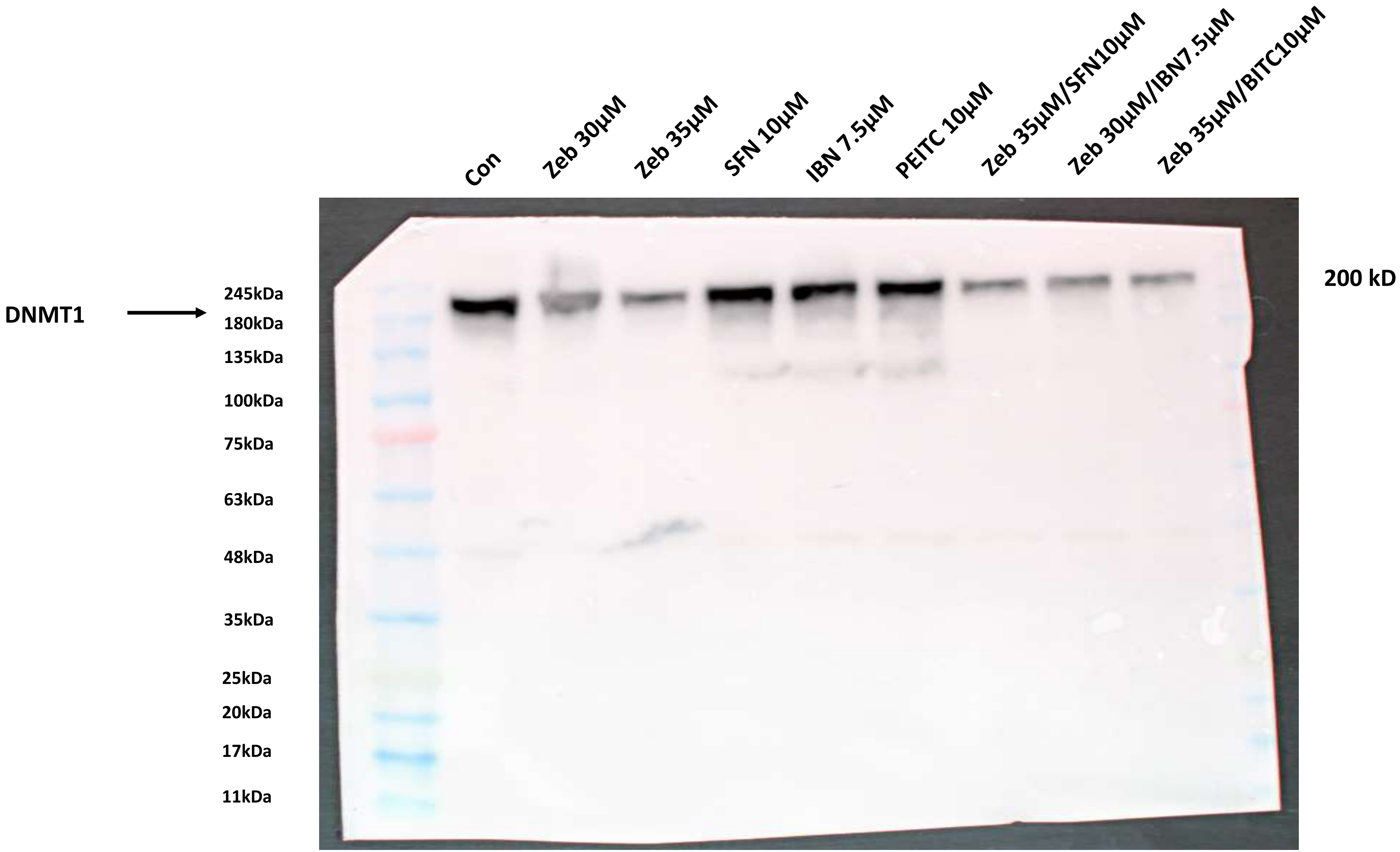

Con
Zeb 30μM
Zeb 35μM
SFN 10μM
IBN 7.5μM
PEITC 10μM
Zeb 35μM/SFN10μM
Zeb 30μM/IBN7.5μM
Zeb 35μM/BITC10μM
DNMT1
245kDa
180kDa
135kDa
100kDa
75kDa
63kDa
48kDa
35kDa
25kDa
20kDa
17kDa
11kDa
200 kD

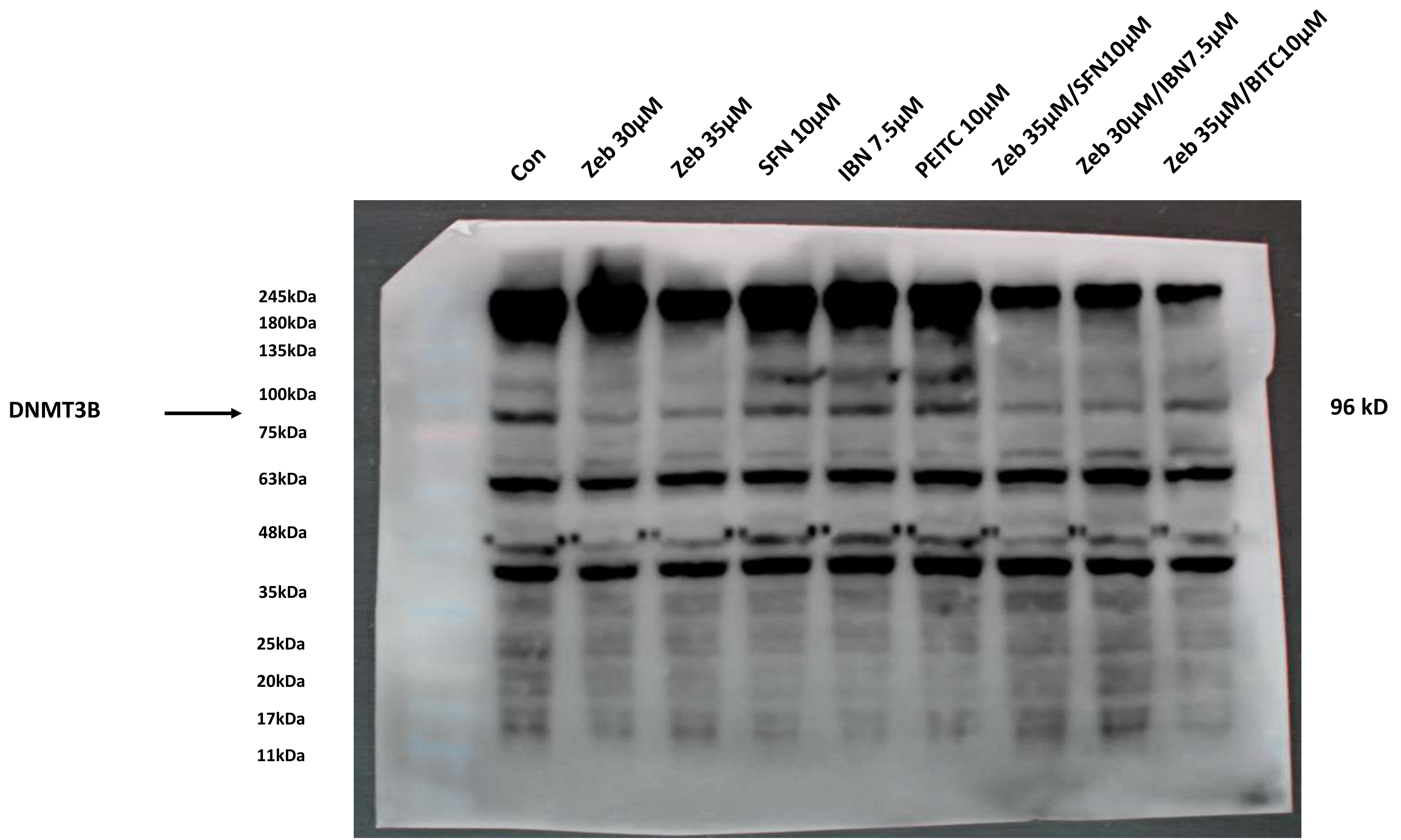
Con
Zeb 30µM
Zeb 35µM
SFN 10µM
IBN 7.5µM
PEITC 10µM
Zeb 35µM/SFN10µM
Zeb 30µM/IBN7.5µM
Zeb 35µM/BITC10µM
245kDa
180kDa
135kDa
100kDa
75kDa
63kDa
48kDa
35kDa
25kDa
20kDa
17kDa
11kDa
DNMT3B
96 kD

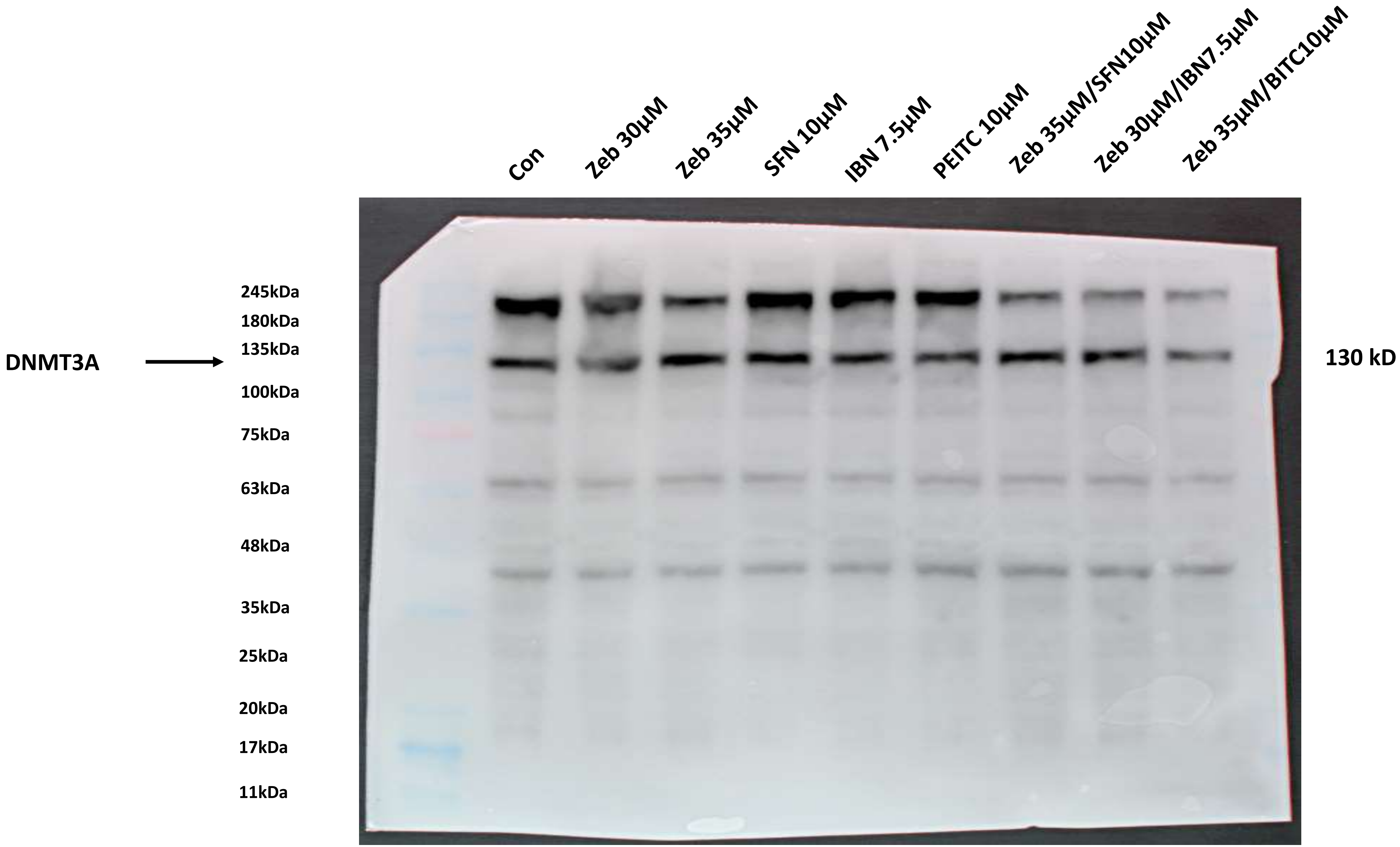
Con
Zeb 30μM
Zeb 35μM
SFN 10μM
IBN 7.5μM
PEITC 10μM
Zeb 35μM/SFN10μM
Zeb 30μM/IBN7.5μM
Zeb 35μM/BITC10μM
DNMT3A
245kDa
180kDa
135kDa
100kDa
75kDa
63kDa
48kDa
35kDa
25kDa
20kDa
17kDa
11kDa
130 kD

Un-cropped western immunoblot membranes in Figure 6

6A.

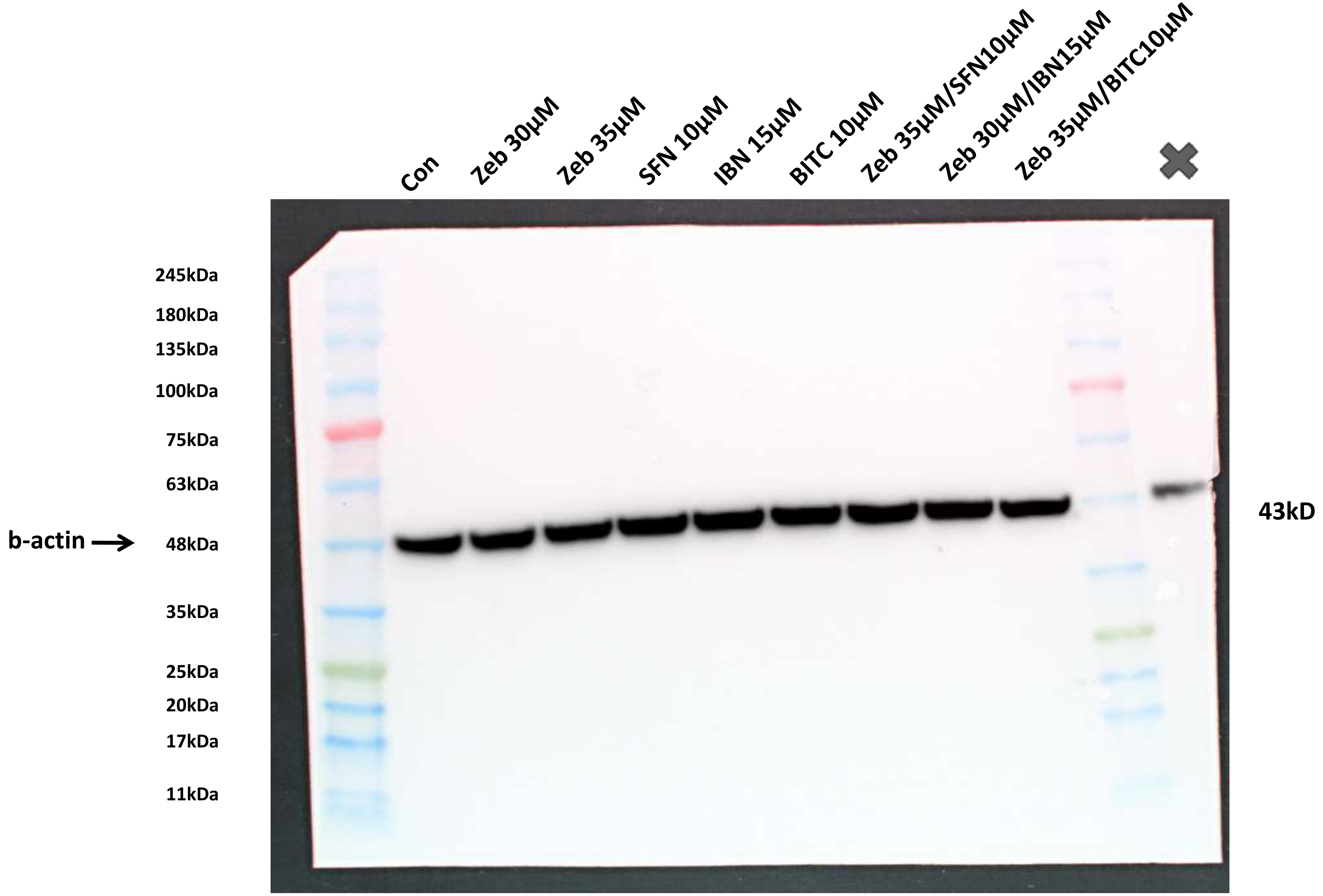

Con
Zeb 30μM
Zeb 35μM
SFN 10μM
IBN 15μM
BITC 10μM
Zeb 35μM/SFN10μM
Zeb 30μM/IBN15μM
Zeb 35μM/BITC10μM
245kDa
180kDa
135kDa
100kDa
75kDa
63kDa
48kDa
35kDa
25kDa
20kDa
17kDa
11kDa
b-actin
43kD

6A.

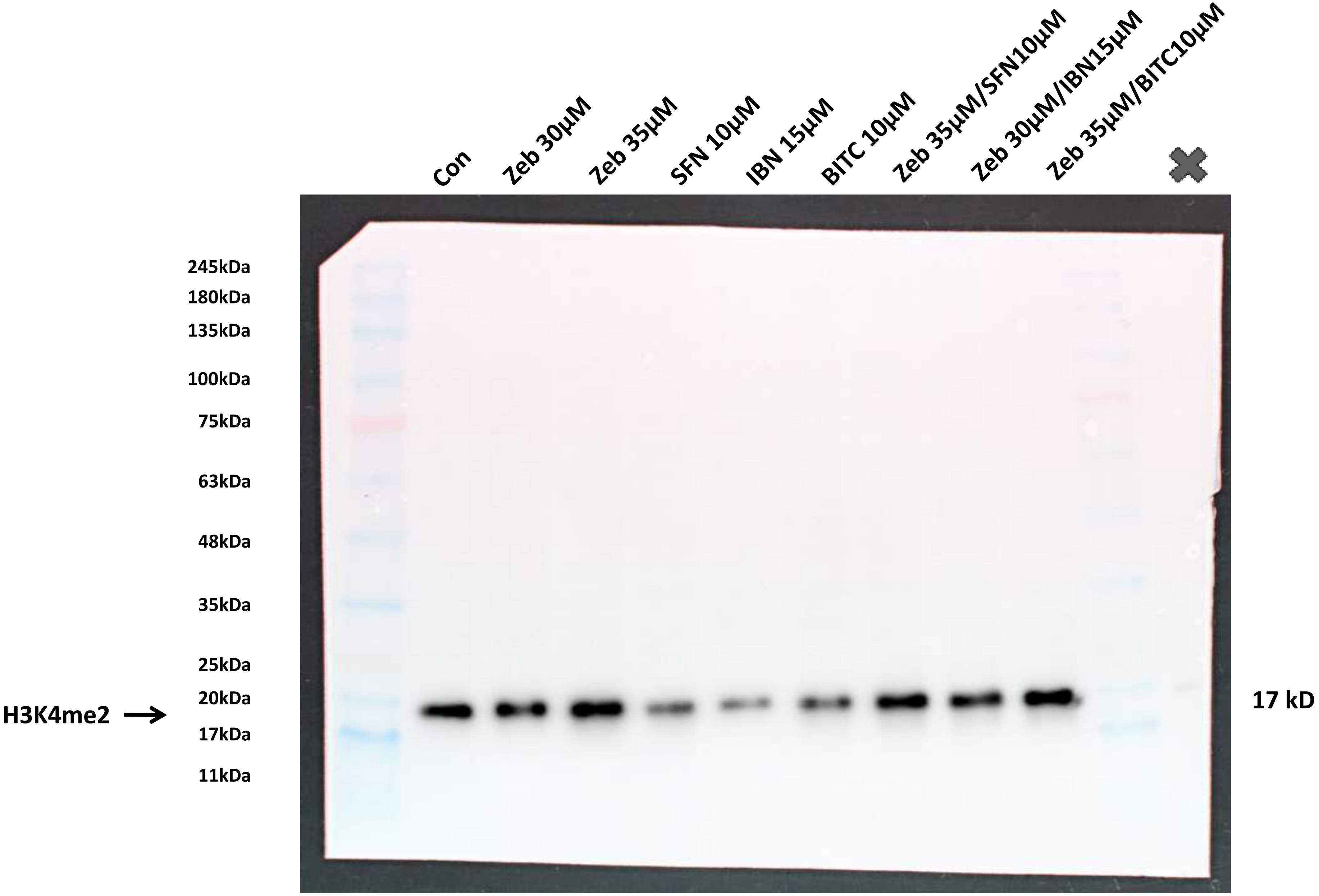
Con
Zeb 30μM
Zeb 35μM
SFN 10μM
IBN 15μM
BITC 10μM
Zeb 35μM/SFN10μM
Zeb 30μM/IBN15μM
Zeb 35μM/BITC10μM
245kDa
180kDa
135kDa
100kDa
75kDa
63kDa
48kDa
35kDa
25kDa
20kDa
17kDa
11kDa
H3K4me2
17 kD

6B.

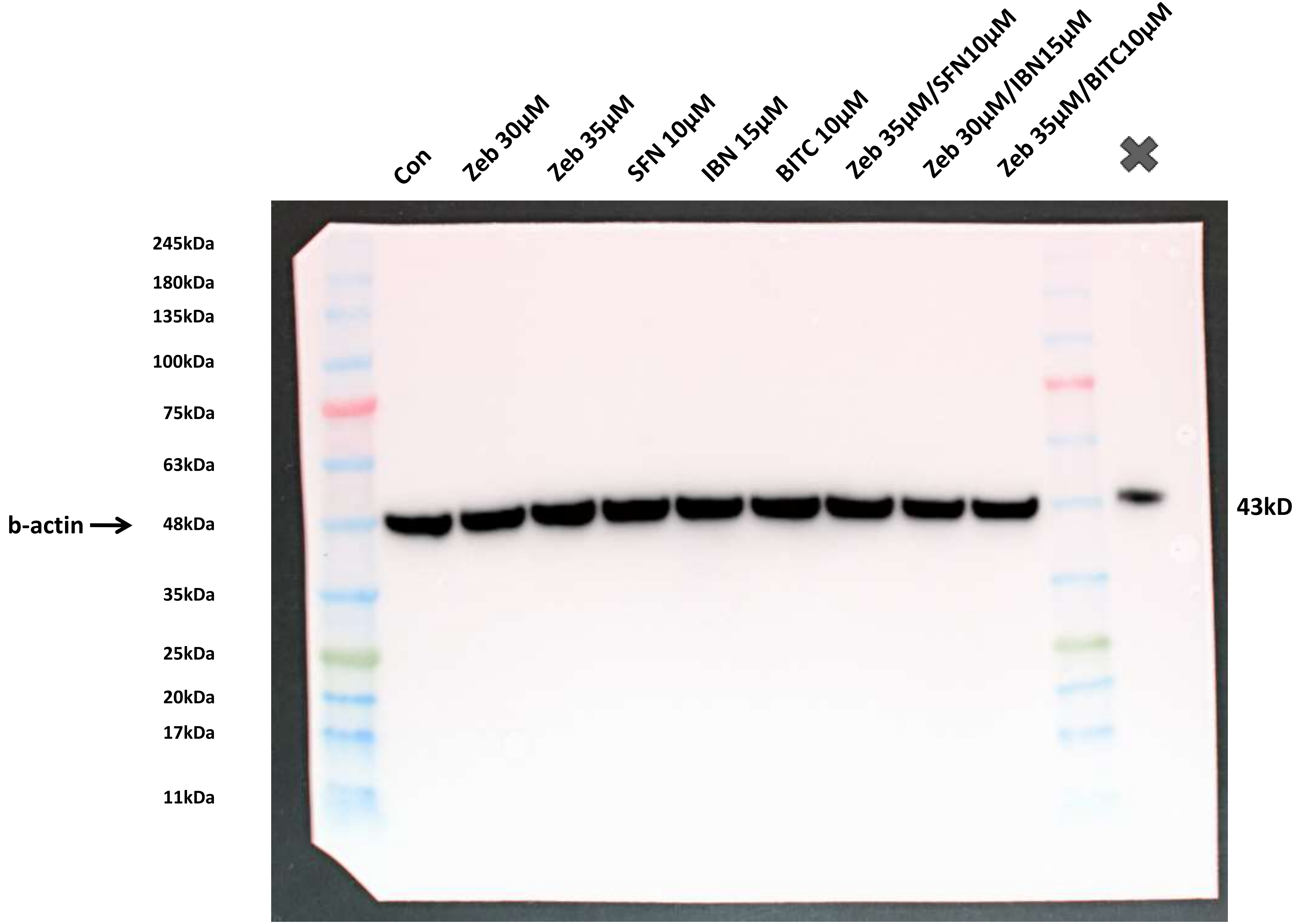
Con
Zeb 30μM
Zeb 35μM
SFN 10μM
IBN 15μM
BITC 10μM
Zeb 35μM/SFN10μM
Zeb 30μM/IBN15μM
Zeb 35μM/BITC10μM
245kDa
180kDa
135kDa
100kDa
75kDa
63kDa
48kDa
35kDa
25kDa
20kDa
17kDa
11kDa
b-actin
43kD

6B.

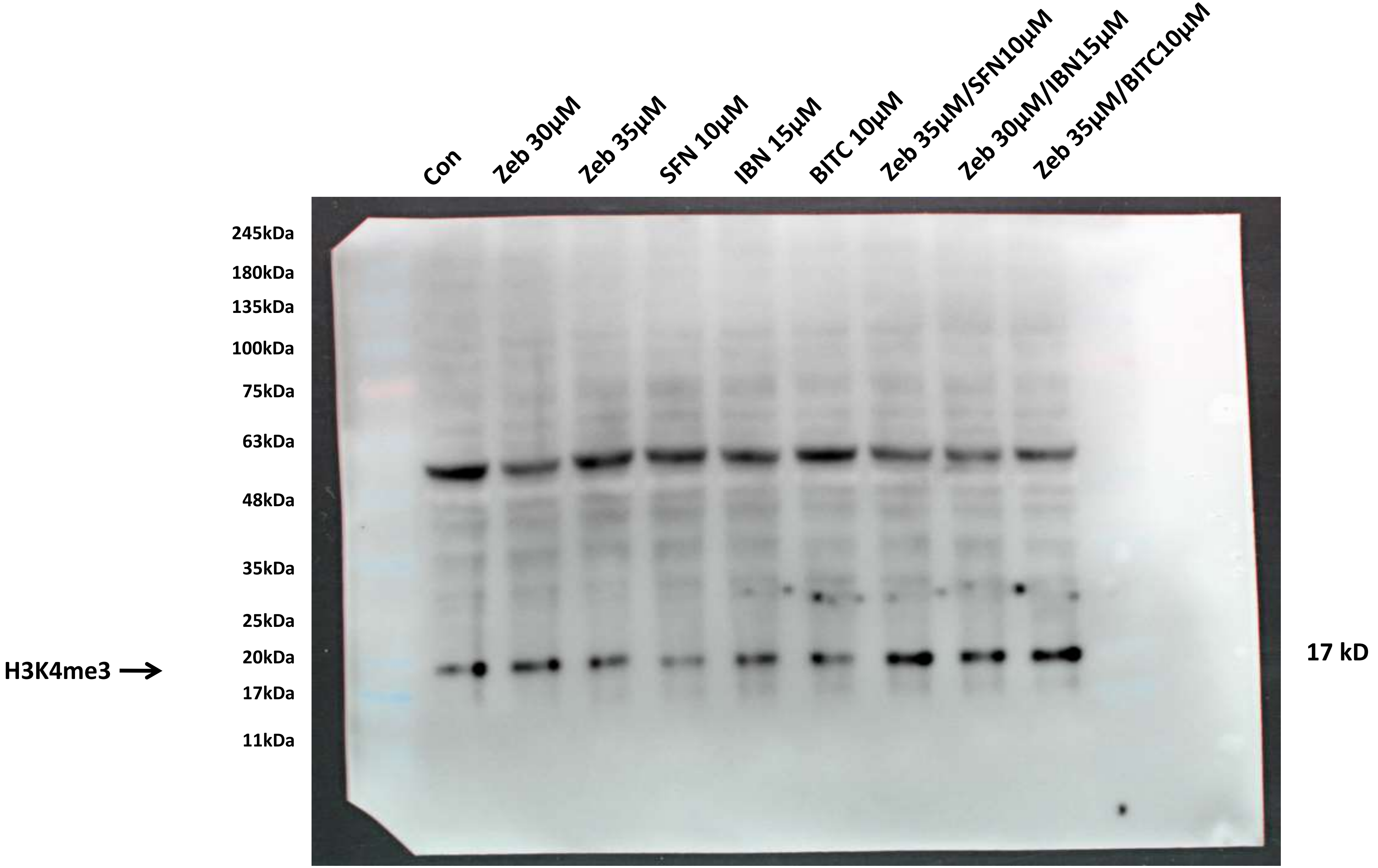

Con
Zeb 30μM
Zeb 35μM
SFN 10μM
IBN 15μM
BITC 10μM
Zeb 35μM/SFN10μM
Zeb 30μM/IBN15μM
Zeb 35μM/BITC10μM
245kDa
180kDa
135kDa
100kDa
75kDa
63kDa
48kDa
35kDa
25kDa
20kDa
17kDa
11kDa
H3K4me3
17 kD

6C.

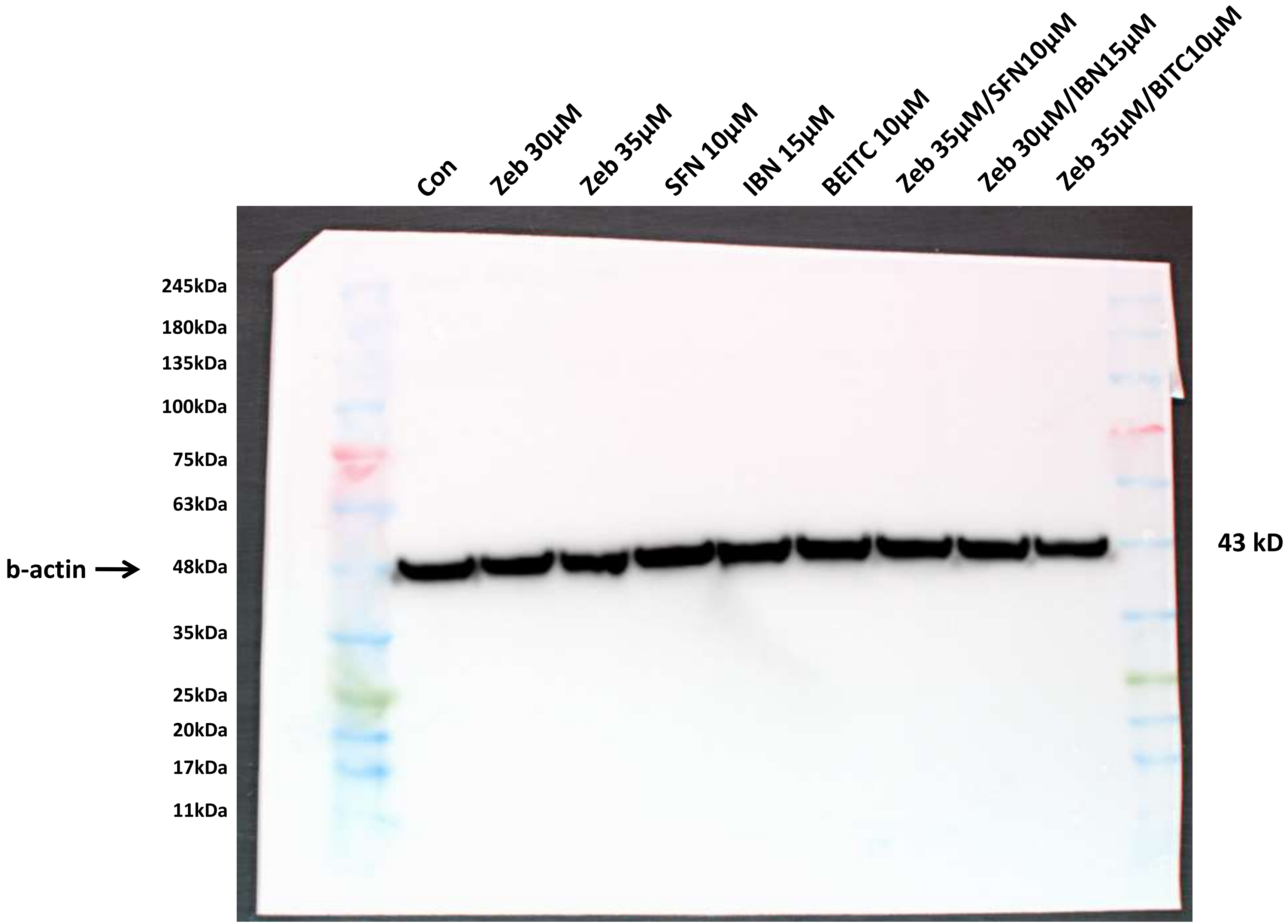

Con
Zeb 30µM
Zeb 35µM
SFN 10µM
IBN 15µM
BEITC 10µM
Zeb 35µM/SFN10µM
Zeb 30µM/IBN15µM
Zeb 35µM/BITC10µM
245kDa
180kDa
135kDa
100kDa
75kDa
63kDa
48kDa
35kDa
25kDa
20kDa
17kDa
11kDa
b-actin
43 kD

6C.

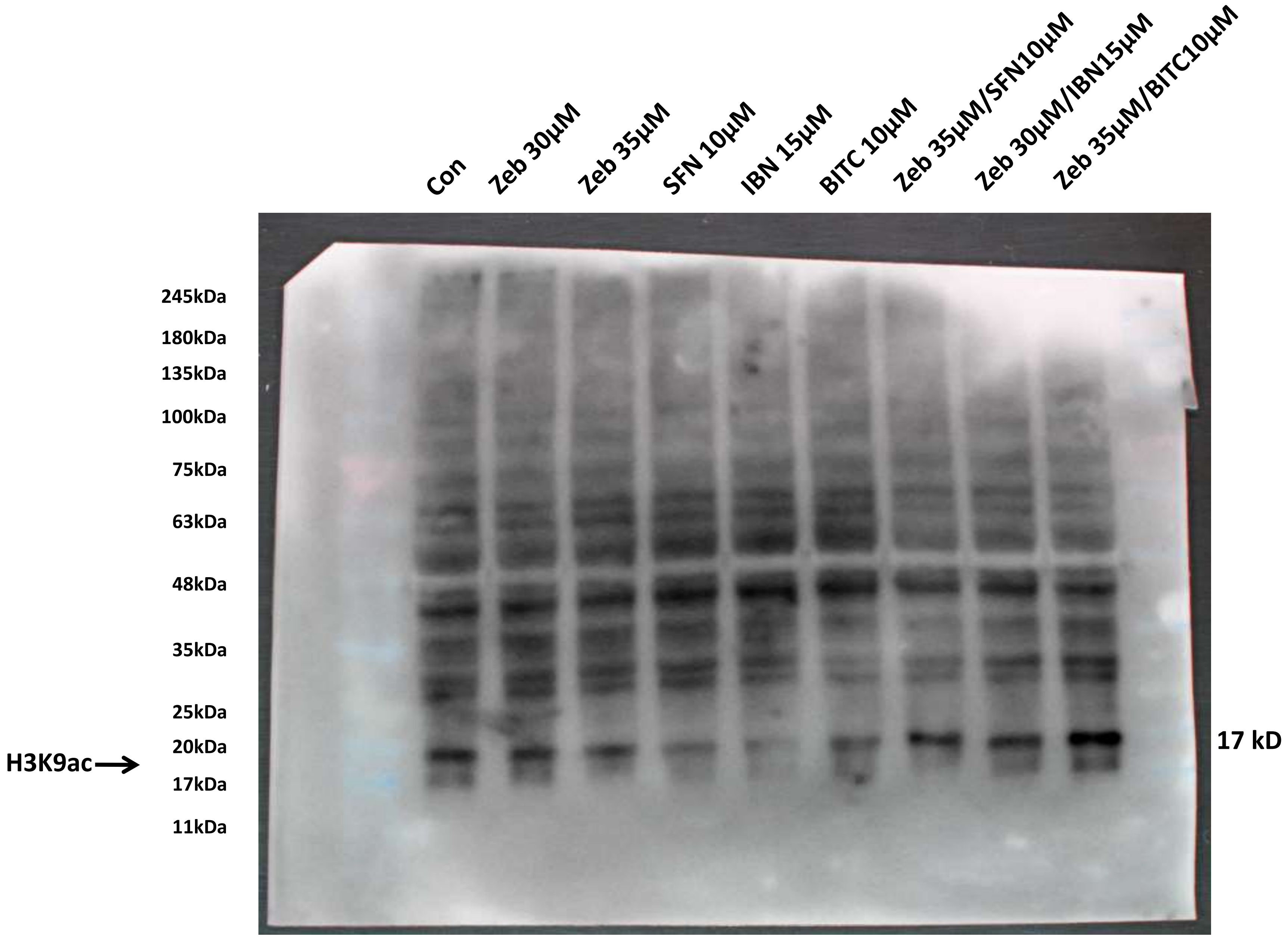
Con
Zeb 30μM
Zeb 35μM
SFN 10μM
IBN 15μM
BITC 10μM
Zeb 35μM/SFN10μM
Zeb 30μM/IBN15μM
Zeb 35μM/BITC10μM
245kDa
180kDa
135kDa
100kDa
75kDa
63kDa
48kDa
35kDa
25kDa
20kDa
17kDa
11kDa
H3K9ac
17 kD

6D.

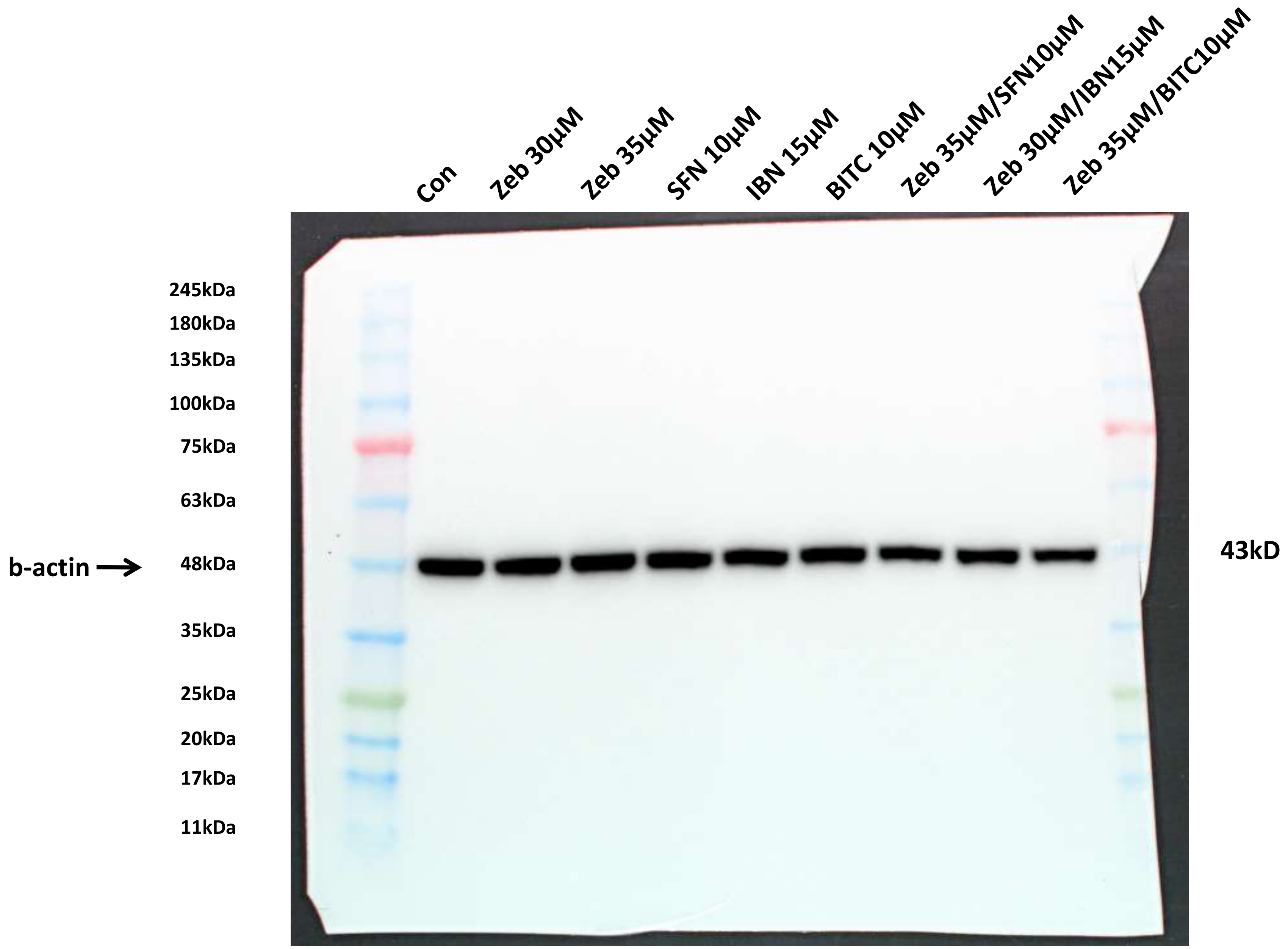
Con
Zeb 30μM
Zeb 35μM
SFN 10μM
IBN 15μM
BITC 10μM
Zeb 35μM/SFN10μM
Zeb 30μM/IBN15μM
Zeb 35μM/BITC10μM
245kDa
180kDa
135kDa
100kDa
75kDa
63kDa
48kDa
35kDa
25kDa
20kDa
17kDa
11kDa
b-actin
43kD

6D.

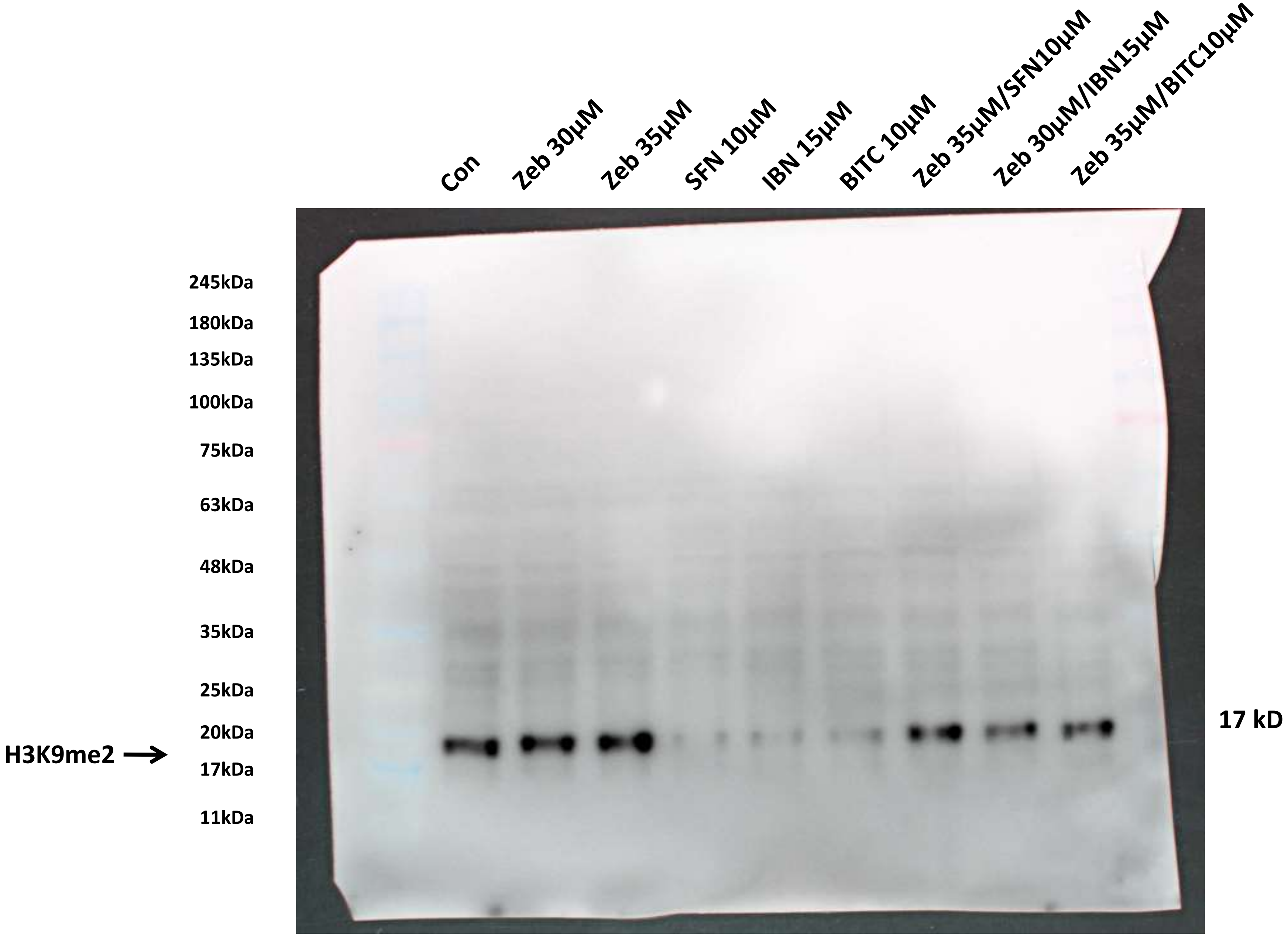

Con
Zeb 30μM
Zeb 35μM
SFN 10μM
IBN 15μM
BITC 10μM
Zeb 35μM/SFN10μM
Zeb 30μM/IBN15μM
Zeb 35μM/BITC10μM
245kDa
180kDa
135kDa
100kDa
75kDa
63kDa
48kDa
35kDa
25kDa
20kDa
17kDa
11kDa
H3K9me2
17 kD

6E.

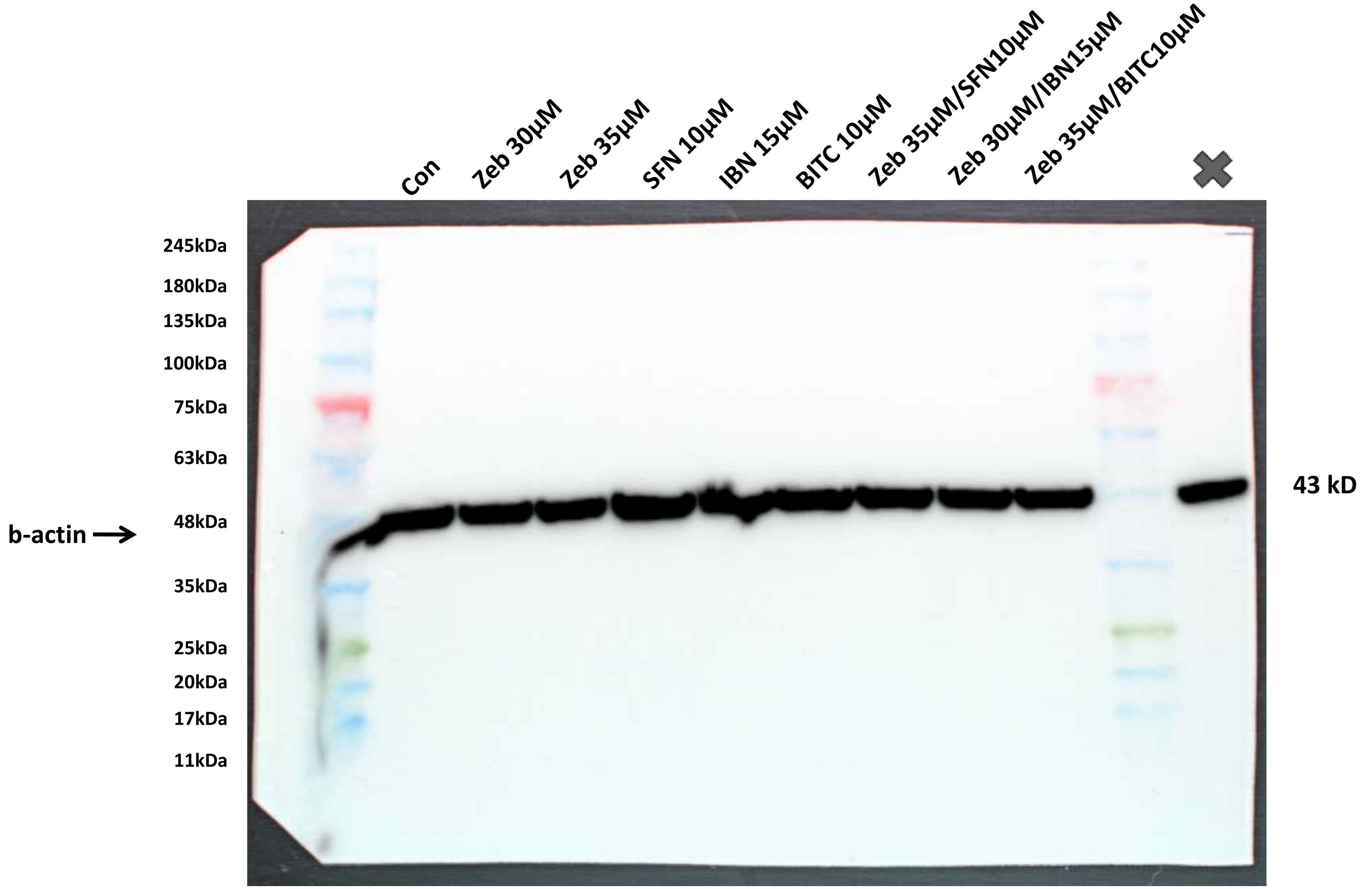

Con
Zeb 30μM
Zeb 35μM
SFN 10μM
IBN 15μM
BITC 10μM
Zeb 35μM/SFN10μM
Zeb 30μM/IBN15μM
Zeb 35μM/BITC10μM
245kDa
180kDa
135kDa
100kDa
75kDa
63kDa
48kDa
35kDa
25kDa
20kDa
17kDa
11kDa
b-actin
43 kD

6E.

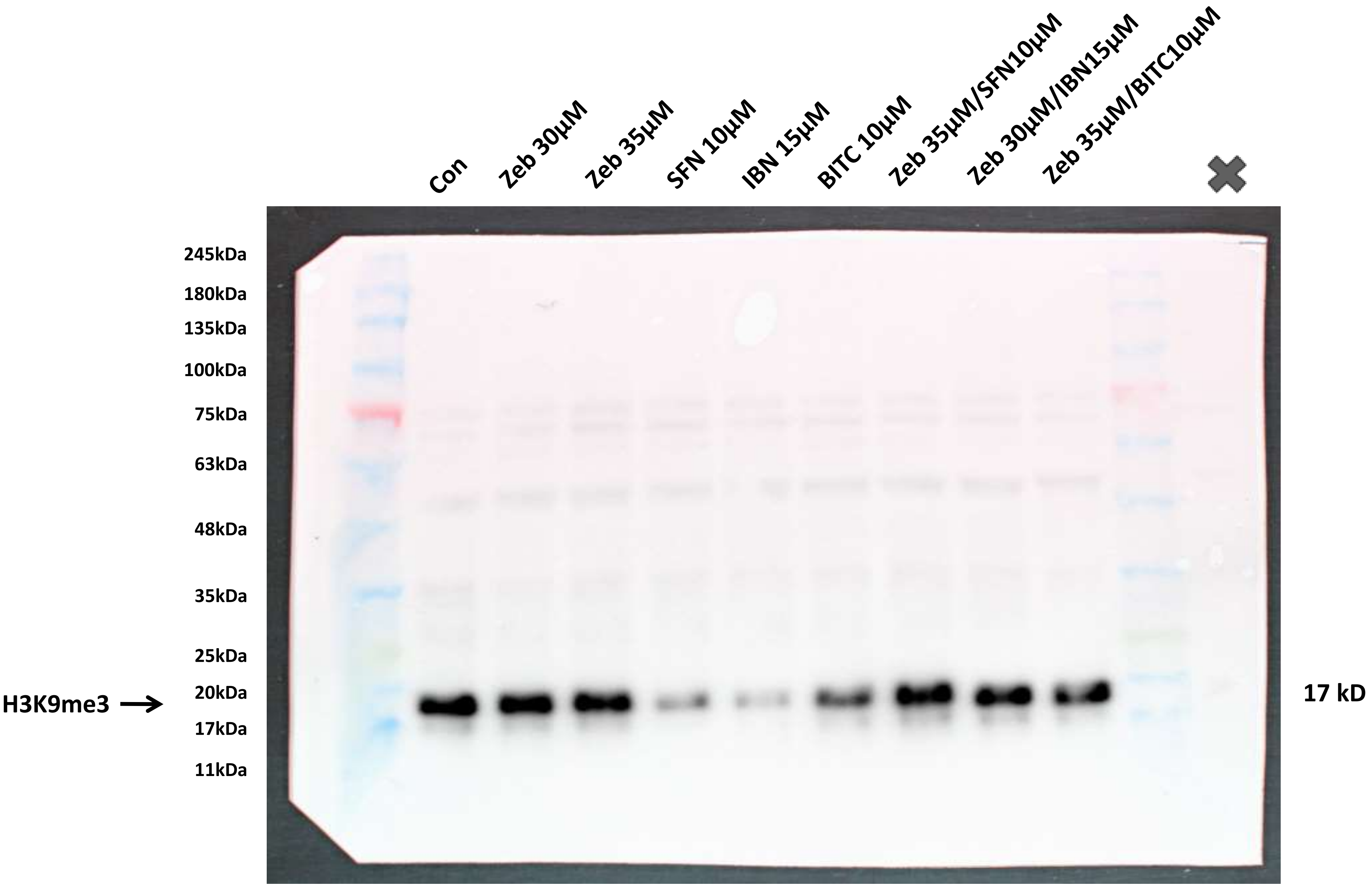

Con
Zeb 30µM
Zeb 35µM
SFN 10µM
IBN 15µM
BITC 10µM
Zeb 35µM/SFN10µM
Zeb 30µM/IBN15µM
Zeb 35µM/BITC10µM
245kDa
180kDa
135kDa
100kDa
75kDa
63kDa
48kDa
35kDa
25kDa
20kDa
17kDa
11kDa
H3K9me3
17 kD

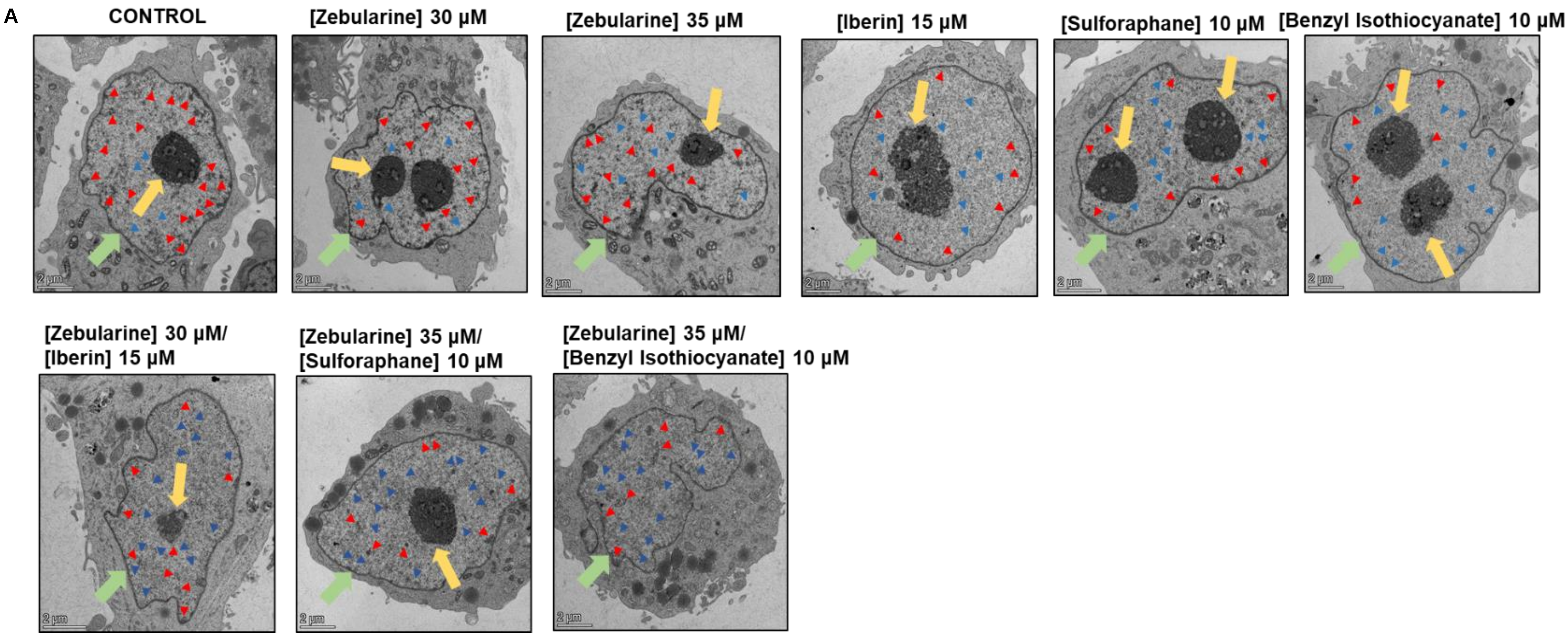
A
CONTROL
[Zebularine] 30 μM
[Zebularine] 35 μM
[Iberin] 15 μM
[Sulforaphane] 10 μM
[Benzyl Isothiocyanate] 10 μM
[Zebularine] 30 μM/
[Iberin] 15 μM
[Zebularine] 35 μM/
[Sulforaphane] 10 μM
[Zebularine] 35 μM/
[Benzyl Isothiocyanate] 10 μM
2 μm

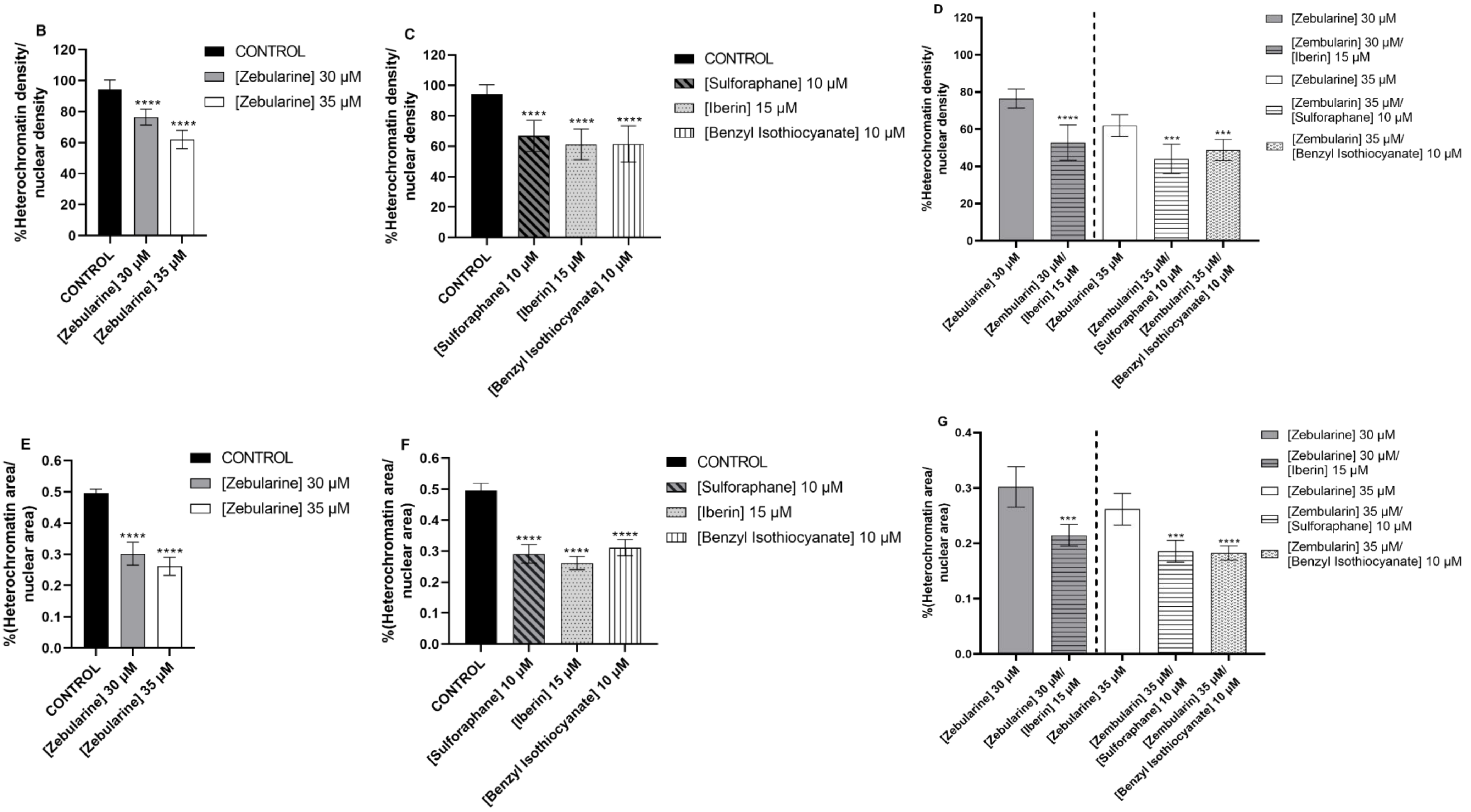
B
%Heterochromatin density/ nuclear density
120
100
80
60
40
20
0
****
****
CONTROL
[Zebularine] 30 μM
[Zebularine] 35 μM
C
%Heterochromatin density/ nuclear density
****
****
****
CONTROL
[Sulforaphane] 10 μM
[Iberin] 15 μM
[Benzyl Isothiocyanate] 10 μM
D
%Heterochromatin density/ nuclear density
****
***
***
[Zebularine] 30 μM
[Zembularin] 30 μM/ [Iberin] 15 μM
[Zebularine] 35 μM
[Zembularin] 35 μM/ [Sulforaphane] 10 μM
[Zembularin] 35 μM/ [Benzyl Isothiocyanate] 10 μM
E
%(Heterochromatin area/ nuclear area)
0.6
0.5
0.4
0.3
0.2
0.1
0.0
****
****
CONTROL
[Zebularine] 30 μM
[Zebularine] 35 μM
F
%(Heterochromatin area/ nuclear area)
****
****
****
CONTROL
[Sulforaphane] 10 μM
[Iberin] 15 μM
[Benzyl Isothiocyanate] 10 μM
G
%(Heterochromatin area/ nuclear area)
0.4
0.3
0.2
0.1
0.0
***
***
****
[Zebularine] 30 μM
[Zebularine] 30 μM/ [Iberin] 15 μM
[Zebularine] 35 μM
[Zembularin] 35 μM/ [Sulforaphane] 10 μM
[Zembularin] 35 μM/ [Benzyl Isothiocyanate] 10 μM

Figure 5. The effect of ZEB and each ITC alone or in combined exposures between ZEB with each ITC on chromatin state/organization. Representative electron micrographs obtained from ultra-thin sections (0.1μm) after exposures of A375 cells with either ZEB (30μM/35μM) and each ITC (10μM SFN, 15μM IBN and 10μM BITC) alone, for 72h. In combinatorial exposures, A375 cells were pre-treated with ZEB (30μM/35μM), for 24h, followed by addition of SFN (35μM ZEB / 10μM SFN), IBN (30μM ZEB / 15μM IBN) and BITC (35μM ZEB / 10μM BITC) for an additional 48h (72h total). Red arrows indicate heterochromatin compact sites (**A**) whereas blue arrows point euchromatin clusters. Nucleus and nucleoli are indicated with green and yellow arrows, respectively. Heterochromatin density/nuclear density (% of control) of A375 cells exposed to ZEB or each ITC alone or in combinatorial exposures between ZEN with each ITC (**B, C, D**). Heterochromatin area/ nuclear area (% of control) of A375 cells exposed to ZEB or each ITC alone or in combinatorial exposures between ZEN with each ITC (**E, F, G**). Overall, 20 micrographs were analyzed through ImageJ software and data are expressed as means ± SEM. In brief, nuclear and heterochromatin area and density were measured by analyzing parameters such as: integrated density, min & max grey value, mean grey value and total area. Statistical significance is indicated by *** $p < 0.001$, and **** $p < 0.0001$ relative to corresponding controls.
